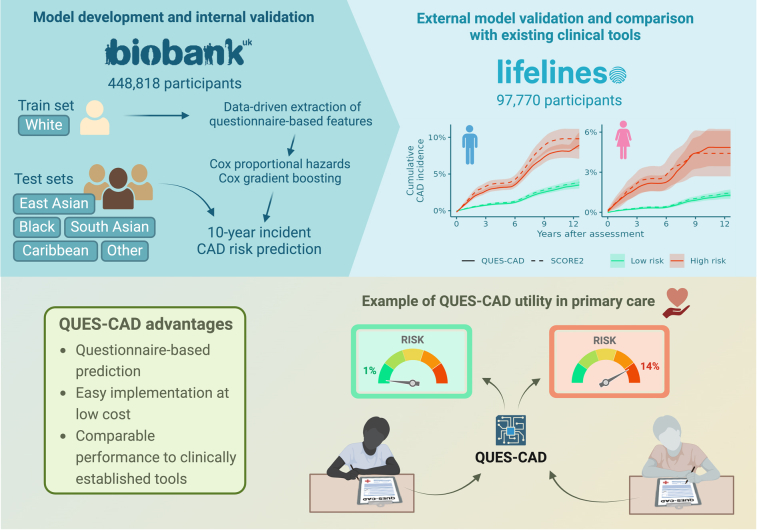# Simplifying coronary artery disease risk stratification: development and validation of a questionnaire-based alternative comparable to clinical risk tools

**DOI:** 10.1016/j.ebiom.2024.105518

**Published:** 2024-12-25

**Authors:** Michail Kokkorakis, Pytrik Folkertsma, Filippos Anagnostakis, Nicole Sirotin, Manyoo Agarwal, Ronney Shantouf, Robert H. Henning, Hanno Pijl, Bruce H.R. Wolffenbuttel, Jeroen J. Bax, Douwe E. Atsma, José Castela Forte, Christos S. Mantzoros, Sipko van Dam

**Affiliations:** a Department of Clinical Pharmacy and Pharmacology, University of Groningen, University Medical Center Groningen, Groningen, Netherlands; b Department of Medicine, Beth Israel Deaconess Medical Center, Harvard Medical School, Boston, MA, USA; c Department of Endocrinology, University of Groningen, University Medical Center Groningen, Groningen, Netherlands; d Center for Biomedical Image Computing and Analytics, University of Pennsylvania, Philadelphia, PA, USA; e Department of Medical and Surgical Sciences, Alma Mater Studiorum University of Bologna, Bologna, Italy; f Department of Preventive Medicine, Cleveland Clinic Abu Dhabi, Abu Dhabi, United Arab Emirates; g Heart, Vascular and Thoracic Institute, Cleveland Clinic Abu Dhabi, Abu Dhabi, United Arab Emirates; h Department of Endocrinology, Leiden University Medical Center, Leiden, Netherlands; i Department of Cardiology, Leiden University Medical Center, Leiden, Netherlands; j National eHealth Living Lab, Leiden, Netherlands; k Department of Design, Organization and Strategy, Faculty of Industrial Design Engineering, Delft University of Technology, Delft, Netherlands; l Boston VA Healthcare System, Harvard Medical School, Boston, MA, USA

**Keywords:** Coronary artery disease, Machine learning, Data-driven prediction, Risk stratification, Discriminative abilities, Population screening

## Abstract

**Background:**

Coronary artery disease (CAD) comprises one of the leading causes of morbidity and mortality both in the European population and globally. All established clinical risk stratification scores and models require blood lipids and physical measurements. The latest reports of the European Commission suggest that attracting health professionals to collect these data can be challenging, both from a logistic and cost perspective, which limits the usefulness of established models and makes them unsuitable for population-wide screening in resource-limited settings, i.e., rural areas. Therefore, the aim of this study was to develop and externally validate a questionnaire-based risk stratification model on a population scale at minimal cost, i.e., the Questionnaire-Based Evaluation for Estimating Coronary Artery Disease (QUES-CAD) to stratify the 10-year incidence of coronary artery disease.

**Methods:**

Cox proportional hazards (CoxPH) and Cox gradient boosting (CoxGBT) models were trained with 10-fold cross-validation using combinations of ten questionnaire variables on the White population of the UK Biobank (n = 448,818) and internally validated the models in all ethnic minorities (n = 27,433). The Lifelines cohort was employed as an independent external validation population (n = 97,770). Additionally, we compared QUES-CAD's performance, containing only questionnaire variables, to clinically established risk prediction tools, i.e., Framingham Coronary Heart Disease Risk Score, American College of Cardiology/American Heart Association pooled cohort equation, World Health Organization cardiovascular disease risk charts, and Systematic Coronary Risk Estimation 2 (SCORE2). We conducted partial log-likelihood ratio (PLR) tests and C-index comparisons between QUES-CAD and established clinical prediction models.

**Findings:**

In the external validation set, QUES-CAD exhibited C-index values of CoxPH: 0.692 (95% Confidence Interval [CI]: 0.673–0.71) and CoxGBT: 0.699 (95% CI: 0.681–0.717) for the male population and CoxPH: 0.771 (95% CI: 0.748–0.794) and CoxGBT: 0.759 (95% CI: 0.736–0.783) for the female population. The addition of measurement-based variables and variables that require a prior medical examination (i.e., insulin use, number of treatments/medications taken, prevalent cardiovascular disease [other than CAD, and stroke diagnosed by a doctor]) and the further addition of biomarkers/other measurements (i.e., high-density lipoprotein [HDL] cholesterol, total cholesterol, and glycated haemoglobin) did not significantly improve QUES-CAD's performance in most instances. C-index comparisons and PLR tests showed that QUES-CAD performs and fits the data at least as well as the clinical prediction models.

**Interpretation:**

QUES-CAD performs comparably to established clinical prediction models and enables a population-wide identification of high-risk individuals for CAD. The model developed and validated herein relies solely on ten questionnaire variables, overcoming the limitations of existing models that depend on physical measurements or biomarkers.

**Funding:**

10.13039/501100005075University Medical Center Groningen.

**Research in context**

**Evidence before this study**

Coronary artery disease (CAD) is a highly prevalent condition and comprises one of the leading causes of morbidity and mortality both in the European population and globally. All clinical risk scores and models require data from laboratory analyses (blood lipids) and physical measurements (among others, blood pressure). This is a considerable burden since, according to the latest reports from the European Commission, attracting and obtaining health professionals to collect these data can be challenging, both from a logistic and cost perspective, limiting the usefulness of the currently available models, especially for population-wide risk stratification in resource-limited settings, i.e., rural areas. Additionally, even though Europe is becoming increasingly ethnically diverse, there is insufficient validation of the clinical risk prediction models among ethnic minorities of European cohorts, which present higher rates of conventional CAD risk factors, differing treatment response rates, excess CAD-related morbidity, mortality, and overall poor quality of life.

**Added value of this study**

Questionnaire-Based Evaluation for Estimating Coronary Artery Disease (QUES-CAD) is a novel Machine Learning questionnaire-based risk stratification tool that forecasts the 10-year incidence of CAD and performs comparable to established clinical risk scores, reflecting lifestyle choices, medical history, and social determinants of health.

**Implications of all the available evidence**

QUES-CAD illustrated risk stratification abilities and performs comparably to established clinical risk prediction tools, by detecting high-risk groups in the population that experience the highest incident rates of CAD, suggesting the potential utility for population-wide screening and identification of populations that might benefit from preventive interventions in a cost-effective and scalable manner.

## Introduction

Coronary artery disease (CAD) is the most common type of heart disease and constitutes one of the leading causes of death globally, with a prevalence of over 7%.^1^ In addition to its significant mortality, CAD is also estimated to account for significant morbidity, accounting for 182 million disability-adjusted life years in 2019 and disproportionately affecting ethnic minority populations.^2^ Moreover, long-standing health inequalities and social disparities in Europe have widened the gap in cardiovascular care, which consequently impacts other disease outcomes.^3^^,^^4^ In detail, CAD is especially implicated as a concomitant condition and complication in eminent cardiometabolic epidemics led by obesity, type 2 diabetes, and metabolic dysfunction-associated steatotic liver disease.^5^, ^6^, ^7^, ^8^, ^9^, ^10^, ^11^ Due to its subclinical disease progression, CAD continues to pose a significant challenge for health systems, which renders crucial the need to propose effective policies to narrow the aforementioned gap.^12^ Primary prevention and novel large-scale population screening programs could help reduce the premature mortality and burden associated with CAD.^12^^,^^13^

Identifying traditional risk factors of CAD has led to the development of scoring algorithms that stratify patients for the risk of incident CAD.^14^ Notably, several risk assessment tools have reached clinical significance and have been included to position themselves in the current cardiovascular disease (CVD) prevention guidelines.^14^ Specifically, the American Heart Association (AHA) recommends using the Framingham Coronary Heart Disease Risk Score (FRS) and the American College of Cardiology/AHA pooled cohort equation (ACC/AHA PCE) as first-line risk assessment tools for incident CVD.^15^^,^^16^ Even though ACC/AHA PCE are currently the most widely recommended screening tool by the AHA, the Predicting Risk of Cardiovascular Disease Events (PREVENT) risk equations are now being introduced into practice as a potential replacement.^17^ Similarly, the European Society of Cardiology suggests using Systematic Coronary Risk Estimation 2 (SCORE2) for the same outcomes.^18^ Recently, revised World Health Organization (WHO) cardiovascular disease risk prediction models were developed, targeting global implementation, particularly in middle- and low-income countries.^19^

A recent report on inequalities in access to healthcare in 35 European countries identifies the inadequate availability of services, particularly in rural areas, as a major challenge.^20^ All the above-mentioned models require data from laboratory analyses (blood lipids) and physical measurements (systolic blood pressure). Therefore, obtaining these data can sometimes be challenging, both from a logistic and cost perspective, limiting the use of these models for population-wide risk stratification. Even though Europe is becoming increasingly ethnically diverse, there is overall insufficient validation of the above prediction models among ethnic minorities in European patient cohorts, which present higher rates of conventional CAD risk factors, differing treatment response rates, excess CAD-related morbidity, all-cause mortality, and overall poor quality of life.^21^, ^22^, ^23^, ^24^ Hence, there is a need for accurate prognostic tools that do not rely on physical or blood chemistry data, can be deployed in a scalable and cost-efficient way, and are validated in a large multi-ethnic population.^25^ This is particularly relevant in resource-limited settings, while sex-specific CAD risk assessment models have yet to receive the attention needed despite large risk and performance differences between female and male populations.

The aim of the current study is to develop and validate prognostic questionnaire-based models to stratify CAD risk (Questionnaire-Based Evaluation for Estimating Coronary Artery Disease [QUES-CAD]) in two independent European biobanks across various ethnic populations.

## Methods

### Study population

The UK Biobank is the largest longitudinal population-based cohort and includes 502,359 individuals aged 37–73 years recruited between 2006 and 2010, with follow-up data until October 2022, ranging between 12 and 17 years after the initial assessment with a median follow-up time of 14 years (Supplementary Fig. S1A).^26^ The Ethics and Guidance Council (http://www.ukbiobank.ac.uk/ethics) oversees ethical practices for the UK Biobank. Before enrolment, all individuals provided informed consent. Lifelines, here employed as a validation cohort, is a multi-disciplinary prospective population-based cohort study examining in a unique three-generation design the health and health-related behaviours of 167,729 persons living in the North of the Netherlands. It employs a broad range of investigative procedures in assessing the biomedical, socio-demographic, behavioural, physical, and psychological factors that contribute to the health and disease of the general population, with a special focus on multi-morbidity and complex genetics. Lifelines comprises data from 168,205 people aged 0 to 93 years, gathered between 2006 and 2013.^27^^,^^28^ Before enrolment, each participant gave written informed consent. A detailed summary of the gathered data can be found in the respective feature catalogues: https://biobank.ndph.ox.ac.uk/showcase/catalogs.cgi and https://data-catalogue.lifelines.nl/. Follow-up times for Lifelines differ per individual, ranging between 0 and 15 years, with the median follow-up time being five years (Supplementary Fig. S1B).

In both study populations, we only included individuals aged ≥40 years.

### Assessment of outcomes

In the UK Biobank, CAD diagnosis was assigned based on ICD-9 codes (36, 410, 411, 412, 414, 429), ICD-10 codes (I21, I22, I23, I24.1, and I25.2), OPCS-4 codes (K401, K402, K403, K404, K411, K412, K413, K414, K451, K452, K453, K454, K45.5, K491, K492, K498, K499, K502, K751, K752, K753, K754, K758, and K759), and operation codes (UK Biobank field: 20004, codes 1070 and 1095); and “heart attack diagnosed by a doctor” (UK Biobank field: 6150, code 1), while controls were identified by the absence of all aforementioned diagnoses and procedure codes. Supplementary Tables S1A and S1B provide the data associated with the patient's age at the time of diagnosis, which were employed to calculate the years until diagnosis from the initial assessment. In cases where multiple ages of diagnosis were reported, the lowest reported age was used. All cases diagnosed before their assessment centre visit were then annotated as prevalent cases, while cases diagnosed after their assessment were annotated as incident cases.

In Lifelines, prevalent and incident CAD were annotated based on self-reported myocardial infarction (assessments 1A, 1B, 1C, 2A, 3A, and 3B), percutaneous coronary intervention (PCI) (assessments 1A, 1B, 1C, 3A, and 3B), and open self-reported diseases (1A, 1B, 1C, 2A, and 3A). The age of diagnosis was not asked during follow-up; therefore, we estimated the age of diagnosis for every incident case by taking the mean of the age the participant had at the assessment reporting a CAD diagnosis and the age of the participant at the previous assessment.^29^

All participants diagnosed with CAD at baseline were excluded from the datasets.

### Predictors

We used a data-driven approach to select the best predictive features. First, all categorical features were transformed to one-hot encoding, maintaining their numerical format. Due to the large number of candidate features in the questionnaire pool, we performed a priori feature selection, starting with an initial list containing all features and sub-selecting those with an absolute correlation greater than 0.01 to the target outcome. Then, the final feature selection was performed by iteratively extracting the top correlated feature and removing its variance from the rest of the features until ten variables were selected. To facilitate external validation, we mapped the input features from the UK Biobank to their associated or closest available Lifelines feature (Supplementary Table S2). We integrated these variables into the existing variable pool to determine whether including physical measurement and biomarker variables enhanced the model's performance. Subsequently, we conducted feature selection and retrained the model to evaluate its performance with the augmented feature set.

### Model development

Using self-reported questionnaire features, we built sex-specific risk stratification models for incident CAD across all ethnicities of the UK Biobank and externally in Lifelines. Self-reported ethnicity was extracted from the UK Biobank, and participants were divided into six different ethnicities (White, South Asian, Caribbean, East Asian, Black, and Other) based on self-perceived ethnicity (Supplementary Table S3). Models were trained on the White population and tested on the Ethnic minorities and Lifelines. Risk probabilities for White individuals themselves were obtained with 10-fold cross-validation.^29^ We used the Python package lifelines (version 0.29.0) to train Cox proportional hazards (CoxPH) regression models with default settings as a baseline model. The time variable was constructed as follows: for cases, the number of years until the event since the baseline assessment was used, while for controls, the years between the baseline assessment and the maximum follow-up date of October 2022 was used unless the participant had deceased prior to that point; in that case, the number of years from the baseline assessment to the date of death was used. Additionally, we trained Cox gradient boosting (CoxGBT) survival models using the Python library scikit-survival (version 0.23.0).^30^ During the training process, we subsampled controls to two times the number of cases in order to speed up training time. The parameters used for training the gradient boosted survival models are described in Supplementary Table S4.

To ensure consistency, all input features were normalised prior to model training using a scaling reference generated on the training dataset by Sklearn's StandardScaler, which was then used to normalise features of both training and test datasets.

### Handling of missing data

To ensure that our model inputs were complete, we excluded participants with missing values. This approach was expected to provide more accurate model performance since it avoids introducing potential biases from imputed data. However, as this approach decreases the sample size and the group sizes of some UK Biobank minorities are relatively small, we also trained the models while imputing missing values based on the train set using Sklearn's iterative imputer. This approach allowed us to perform the risk stratification analysis (Kaplan–Meier analysis; described below) with more subjects, though at the potential cost of reduced discriminative accuracy.

### Implementation of existing models

We validated FRS with and without laboratory parameters, both laboratory and non-laboratory-based models developed by the revised WHO cardiovascular disease risk prediction approaches, the ACC/AHA PCE for atherosclerotic cardiovascular disease risk estimation score, as well as SCORE2.^15^^,^^16^^,^^18^^,^^19^^,^^31^

Risk probabilities for FRS and ACC/AHA PCE were calculated using the R package Cvrisk (version 1.1.0). For SCORE2 and WHO, risk probabilities were calculated as described elsewhere.^18^, ^19^ We also included age as a standalone predictor (as a reference point).

### Statistical analysis

The discriminatory performance of the models was evaluated by means of the concordance index (C-index) with a 95% confidence interval (CI) using the survival package (version 3.5.7). We performed partial log-likelihood ratio (PLR) tests between our models and the included existing models using the nonnestcox package (version 0.0.0) to compare the goodness of fit of the models, as well as C-index tests using the compareC package (version 1.3.2) to compare the discriminatory performance of the models. Significance thresholds were adjusted using Bonferroni correction.

We performed a more in-depth comparison between the QUES-CAD models and SCORE2. Typically, a SCORE2 threshold of ≥10% risk is used to classify individuals aged 50–69 years into very high- and lower-risk categories (https://www.escardio.org/Journals/E-Journal-of-Cardiology-Practice/Volume-22/rapid-online-self-assessment-of-individual-risk-for-cardiovascular-events-in-a). However, as some of the minority groups in the UK Biobank are relatively small, we opted for a lower SCORE2 cutoff of 0.075 (which is typically used to identify individuals at very high risk aged 40–49 years) to classify individuals into very high- and lower-risk groups to increase the confidence of the statistical analysis in the high-risk group. To ensure a meaningful, interpretable, and fair comparison, we extracted the probability thresholds for our models that returned the same group size for the White ethnic group as SCORE2's threshold of 0.075 did. This approach accounts for differences in model calibration, which reflect their development datasets and can skew direct comparisons at fixed probability thresholds. By aligning group sizes, we ensured that performance metrics, such as sensitivity and specificity, reflected the models' discriminatory capabilities rather than calibration-driven differences. We used the survivalROC package (version 0.3.1) to calculate the time-dependent sensitivity and specificity of our models for stratifying 10-year CAD risk. Overall, the 10-year incidence was estimated using the Kaplan–Meier method from the survival package. Time-dependent positive predictive value (PPV) and negative predictive value (NPV) were derived from the sensitivity, specificity, and incidence (Supplementary Table S5). 95% CI were calculated using 100 bootstraps. The R package ggsurvfit (version 1.0.0) was used to visualise cumulative incidence over a 15-year follow-up period. All statistical analyses were conducted using R version 3.6.1 and for the model training analyses Python version 3.9 was used.

### Role of the funding source

The funder had no role in study design, data collection and analysis, decision to publish, or preparation of the manuscript.

### Ethics

UK Biobank has approval from the North West Multi-centre Research Ethics Committee (MREC) as a Research Tissue Bank (RTB) approval. This approval means that researchers do not require separate ethical clearance and can operate under the RTB approval. The Lifelines protocol was approved by the University Medical Center Groningen Medical Ethical Committee under number 2007/152. All participants signed an informed consent form. No participants were re-contacted during this project.

## Results

### Baseline characteristics

**Fig. 1:**
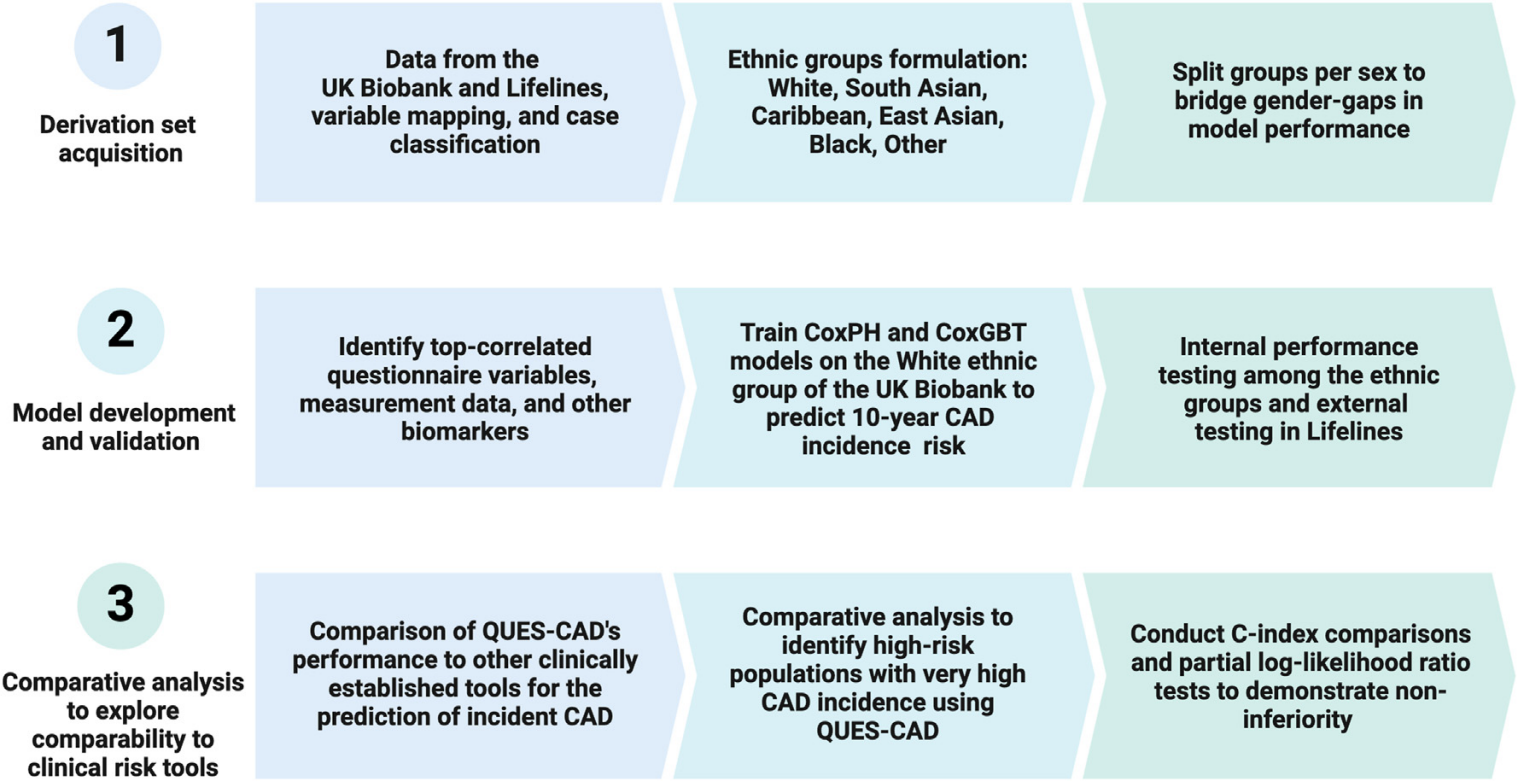
A brief overview of the methodology used in this study. Abbreviations: CAD, coronary artery disease; QUES-CAD, Questionnaire-Based Evaluation for Estimating Coronary Artery Disease; CoxPH, Cox proportional hazards; CoxGBT, Cox gradient boosting. Created with BioRender.com.

A brief overview of the methods is presented in Fig. 1, and detailed baseline characteristics are shown in Table 1, Table 2. This study population included 476,251 from the UK Biobank and 97,770 from Lifelines to train, validate, and externally test the machine learning (ML) models (Table 1, Table 2). The training set comprised 201,334 White individuals from the UK Biobank for men and 247,484 for women (Table 1, Table 2). The models were tested internally among 12,617 individuals for men and 14,816 for women of five different ethnic backgrounds and externally tested in Lifelines (n = 40,580 for men, n = 57,190 for women) (Table 1, Table 2, Supplementary Table S6).

**Table 1:** Baseline characteristics of the internal and external study of male populations.

Population	White men		South Asian men		Caribbean men		East Asian men		Black men		Other men		Lifelines men	
	Cases	Controls	Cases	Controls	Cases	Controls	Cases	Controls	Cases	Controls	Cases	Controls	Cases	Controls
Total size (n)	11,093	190,241	340	3543	71	1698	77	1709	43	1752	192	3192	869	39,711
**Questionnaire variables**														
Age (years)	62 (9.9)	58.4 (13.3)	58.7 (13)	52.4 (14.5)	57.5 (15.8)	51.4 (12.8)	55.6 (14.8)	51 (14.2)	57.8 (12.9)	49.5 (11.9)	58.8 (11)	53.1 (15.2)	58.3 (16.5)	49.5 (13.2)
Alcohol intake frequency (1: Daily or almost daily, 2: Three or four times a week, 3: Once or twice a week, 4: One to three times a month, 5: Special occasions only, 6: Never)	3 (2)	2 (2)	5 (3)	5 (3)	3 (2)	3 (3)	4 (3)	4 (2)	5 (3)	5 (3)	4 (3)	3 (3)	3 (1)	3 (1)
Average total household income before tax (1: <£18,000, 2: £18,000–£30,999, 3: £31,000–£51,999, 4: ≥152,000)	2 (2)	3 (2)	2 (2)	2 (3)	2 (2)	2 (2)	2 (2)	2 (3)	2 (2)	2 (2)	2 (2)	2 (2)	3 (2)	3 (2)
Aspirin use	28.60%	15.40%	34.40%	17.60%	35.20%	12%	23.40%	12.30%	41.90%	13.80%	24.60%	14.70%	99%	99.60%
Body mass index (kg/m^2^)	28.2 (5.4)	27.2 (5)	27 (5.1)	26.5 (4.7)	28 (5.1)	28 (5.1)	26.8 (5.1)	26 (4.5)	29.1 (5.9)	27.8 (5.1)	28.4 (5.2)	27.4 (5.1)	26.9 (4.2)	26.4 (4.3)
Current smoking	17.30%	11.80%	13.80%	14.10%	23.90%	23.30%	26%	16.60%	18.60%	11.30%	24%	17.80%	21.90%	19.80%
Heart disease of father	34%	27.20%	34.10%	25.70%	17.20%	8.80%	29.70%	19.50%	10.80%	5.80%	27.50%	21.20%	15.10%	7.30%
Heart disease of mother	21.70%	15.40%	19.20%	14.10%	7.40%	8.50%	16%	11.40%	7.70%	4.80%	19.30%	12.90%	31.20%	21.40%
Number of cigarettes currently smoked daily (current cigarette smokers)	20 (8)	15 (10)	10 (8)	10 (8)	15 (7.5)	10 (9)	10 (10.2)	12 (9)	10 (4)	10 (8)	15 (8)	15 (10)	15 (10)	13 (12)
Pack years of smoking	27 (26.9)	21 (23.5)	17.5 (16)	14.7 (15.2)	20.1 (27.8)	14.2 (14.1)	16.8 (18.4)	16.5 (16.7)	17.7 (29.4)	13 (14.1)	20.4 (22.1)	18 (20)	15 (17.4)	12.5 (15.4)
Unable to work because of sickness or disability	7.90%	4%	12.60%	5.50%	16.90%	7.20%	5.20%	4.60%	14%	4.70%	18.10%	6.40%	6.10%	3.20%
Usual walking pace (1: Slow pace, 2: Steady average pace, 3: Brisk pace)	2 (1)	2 (1)	2 (1)	2 (0)	2 (0)	2 (1)	2 (0)	2 (0)	2 (1)	2 (0)	2 (1)	2 (1)	2 (0)	2 (0)
Dentures	25.80%	16.50%	17.10%	9.20%	26.80%	19.60%	14.30%	13.70%	20.90%	9.30%	18.70%	13.90%	22.70%	13.80%
**Measurement-based variables and variables that require prior medical examination**														
Insulin use	3.10%	1.10%	10.90%	2.40%	9.90%	2.90%	5.20%	1.50%	2.30%	2.40%	8.80%	1.80%	5.60%	2.50%
Number of treatments/medications taken	3 (4)	1 (3)	3 (6)	1 (3)	3 (4.5)	1 (3)	2 (5)	1 (3)	4 (4.5)	1 (3)	3 (4)	1 (3)	1 (3)	0 (2)
Prevalent cardiovascular disease (other than coronary artery disease)	20%	6.40%	29.10%	6.90%	16.90%	4.10%	19.50%	4%	25.60%	4.50%	21.40%	5.50%	21.70%	11.70%
Stroke diagnosed by a doctor	3.60%	1.70%	3.80%	1.40%	7%	1.30%	0%	0.70%	2.30%	1.50%	3.50%	0.90%	1.70%	1.10%
Mean systolic blood pressure (mmHg)	143.5 (24.5)	139 (23)	140.8 (22.5)	133.5 (22)	142.5 (23.5)	137 (23)	135 (17.5)	133 (22.5)	143 (22.2)	137 (22.9)	143.5 (23)	135 (23)	137 (20)	130 (18)
Waist-to-hip ratio	1 (0.1)	0.9 (0.1)	1 (0.1)	0.9 (0.1)	0.9 (0.1)	0.9 (0.1)	0.9 (0.1)	0.9 (0.1)	1 (0.1)	0.9 (0.1)	1 (0.1)	0.9 (0.1)	1 (0.1)	1 (0.1)
**Laboratory variables**														
HDL cholesterol (mmol/L)	1.2 (0.4)	1.3 (0.4)	1.1 (0.3)	1.1 (0.3)	1.3 (0.4)	1.3 (0.4)	1.2 (0.3)	1.2 (0.3)	1.3 (0.3)	1.2 (0.4)	1.1 (0.3)	1.2 (0.4)	1.2 (0.3)	1.3 (0.4)
Total cholesterol (mmol/L)	5.5 (1.7)	5.5 (1.5)	5.2 (1.8)	5.3 (1.5)	5.2 (2.1)	5.2 (1.4)	5.5 (2)	5.4 (1.5)	5 (2)	5.1 (1.5)	5.3 (1.6)	5.3 (1.5)	5.6 (1.3)	5.3 (1.2)
Glycated haemoglobin (HbA1c) (mmol/mol)	36.4 (5.9)	35.1 (5.1)	40.6 (15.7)	38 (7.2)	39.6 (8.4)	38.1 (6.5)	38.5 (7.7)	37.1 (6.3)	38.5 (9.7)	37.8 (7.2)	38.8 (8.9)	36.3 (6.3)	39 (5)	38 (5)

Data are presented as the mean (Standard Deviation [SD]) unless otherwise noted. The measurements are presented as median (Interquartile range [IQR]).

**Table 2:** Baseline characteristics of the internal and external study of female populations.

Population	White women		South Asian women		Caribbean women		East Asian women		Black women		Other women		Lifelines women	
	Cases	Controls	Cases	Controls	Cases	Controls	Cases	Controls	Cases	Controls	Cases	Controls	Cases	Controls
n	5321	242,163	116	3390	60	3001	37	2166	27	1880	83	4056	433	56,757
**Questionnaire variables**														
Age (years)	62.9 (8.8)	58 (12.7)	59 (11)	52.8 (13.3)	58.6 (12.4)	50.9 (11.7)	61.5 (7.3)	52.7 (12.8)	57.2 (17.6)	50.5 (12.3)	60.8 (13.3)	53.1 (13.3)	59.3 (16.2)	49.4 (12.8)
Alcohol intake frequency (1: Daily or almost daily, 2: Three or four times a week, 3: Once or twice a week, 4: One to three times a month, 5: Special occasions only, 6: Never)	3 (3)	3 (2)	6 (1)	6 (1)	5 (2)	4 (2)	5 (3)	5 (3)	5 (3)	5 (2)	5 (3)	5 (2)	4 (3)	3 (3)
Average total household income before tax (1: <£18,000, 2: £18,000 –£30,999, 3: £31,000–£51,999, 4: ≥152,000)	2 (2)	2 (1)	2 (2)	2 (2)	1 (1)	2 (2)	2 (1)	2 (3)	1 (1)	2 (2)	2 (2)	2 (2)	2 (2)	3 (2)
Aspirin use	21.70%	9%	31%	11.70%	33.30%	10.50%	27%	7.30%	33.30%	11.90%	24.30%	9.60%	100%	99.90%
Body mass index (kg/m^2^)	27.7 (7.1)	26 (6.2)	28.3 (6.6)	26.7 (6.1)	30.7 (6.7)	28.6 (7.7)	25.7 (5.2)	24.5 (5.4)	31.1 (5.6)	30 (7.5)	28.8 (7)	26.6 (7.1)	26.6 (5.6)	25.5 (5.7)
Current smoking	17.70%	8.70%	3.40%	3%	21.70%	13%	5.40%	6.30%	7.40%	6%	14.90%	10.50%	25.40%	17.10%
Heart disease of father	38.70%	30.50%	39.10%	29.30%	20%	11.80%	41.70%	22.90%	12.50%	7.40%	25.40%	21.90%	20.10%	9.60%
Heart disease of mother	30.50%	20.60%	27.30%	18.20%	13.30%	12.70%	35.10%	15.40%	8%	8.50%	31.40%	16.80%	36.70%	23.60%
Number of cigarettes currently smoked daily (current cigarette smokers)	15 (10)	15 (10)	6 (7.5)	9 (6)	10 (8)	10 (9)	10 (5)	10 (9)	20 (0)	10 (9)	10 (13)	10 (9)	12 (7.5)	10 (9)
Pack years of smoking	24.4 (22.8)	16.8 (19.5)	17 (22.7)	10.1 (12.2)	17.8 (12.2)	11.9 (13.2)	32.6 (14.5)	12.5 (14.2)	12.3 (19.7)	12.6 (15.5)	21.9 (22.6)	13.8 (17.4)	12.8 (18.3)	9 (13.7)
Unable to work because of sickness or disability	7%	3%	11.20%	6.10%	16.70%	6.20%	5.40%	3.10%	14.80%	5.60%	10.80%	5.50%	9.90%	4.40%
Usual walking pace (1: Slow pace, 2: Steady average pace, 3: Brisk pace)	2 (1)	2 (1)	2 (1)	2 (0)	2 (1)	2 (1)	2 (0)	2 (0)	2 (1)	2 (0)	2 (1)	2 (1)	2 (0)	2 (0)
Dentures	30.40%	15.60%	15.50%	8.30%	35%	19.50%	18.90%	16.20%	22.20%	10.10%	18.90%	13.70%	26.60%	12.60%
**Measurement-based variables and variables that require prior medical examination**														
Insulin use	3.30%	0.70%	10.30%	1.80%	15%	1.90%	5.40%	1%	3.70%	1.70%	4.10%	1.30%	5.80%	1.90%
Number of treatments/medications taken	3 (4)	2 (3)	4 (4)	2 (3)	4 (4.5)	2 (3)	4 (3)	1 (3)	4 (4)	2 (3)	2 (6)	2 (3)	2 (4)	1 (2)
Prevalent cardiovascular disease (other than coronary artery disease)	16%	3.60%	25%	5.40%	20%	4.50%	16.20%	2.70%	25.90%	4.90%	18.10%	3.60%	25.20%	13.50%
Stroke diagnosed by a doctor	3.20%	1%	2.60%	1%	1.70%	1.40%	0%	0.50%	7.40%	1%	2.70%	0.80%	2.30%	0.90%
Mean systolic blood pressure (mmHg)	141 (27.5)	132.5 (26)	140.8 (26.8)	130 (26.5)	144.2 (30.1)	132.5 (26.2)	141.5 (21)	128.5 (27)	146 (16.5)	134.5 (27.5)	143.8 (29.5)	129.5 (26.5)	132 (22.2)	123 (21)
Waist-to-hip ratio	0.8 (0.1)	0.8 (0.1)	0.9 (0.1)	0.8 (0.1)	0.9 (0.1)	0.8 (0.1)	0.9 (0.1)	0.8 (0.1)	0.9 (0.1)	0.8 (0.1)	0.9 (0.1)	0.8 (0.1)	0.9 (0.1)	0.9 (0.1)
**Laboratory variables**														
HDL cholesterol (mmol/L)	1.4 (0.5)	1.6 (0.5)	1.2 (0.3)	1.4 (0.4)	1.4 (0.5)	1.5 (0.5)	1.5 (0.4)	1.5 (0.5)	1.3 (0.6)	1.5 (0.5)	1.4 (0.5)	1.5 (0.5)	1.5 (0.5)	1.6 (0.5)
Total cholesterol (mmol/L)	6 (1.8)	5.8 (1.5)	5.4 (1.5)	5.4 (1.4)	5.3 (1.5)	5.3 (1.4)	5.4 (2.1)	5.7 (1.5)	5 (1.2)	5.2 (1.4)	5.9 (1.8)	5.6 (1.5)	5.6 (1.4)	5.2 (1.3)
Glycated haemoglobin (HbA1c) (mmol/mol)	36.7 (5.5)	35.1 (4.8)	42.1 (18.3)	37.8 (6.8)	42.4 (12.6)	37.3 (7.1)	38.8 (8.2)	36.5 (5.9)	42.8 (12.2)	37.1 (6.5)	39.2 (7.8)	36.1 (5.9)	39 (5)	38 (5)

Data are presented as the mean (Standard Deviation [SD]) unless otherwise noted. The measurements are presented as median (Interquartile range [IQR]).

### Performance of questionnaire-based coronary artery disease risk stratification models (QUES-CAD) in comparison to established clinical models

**Fig. 2:**
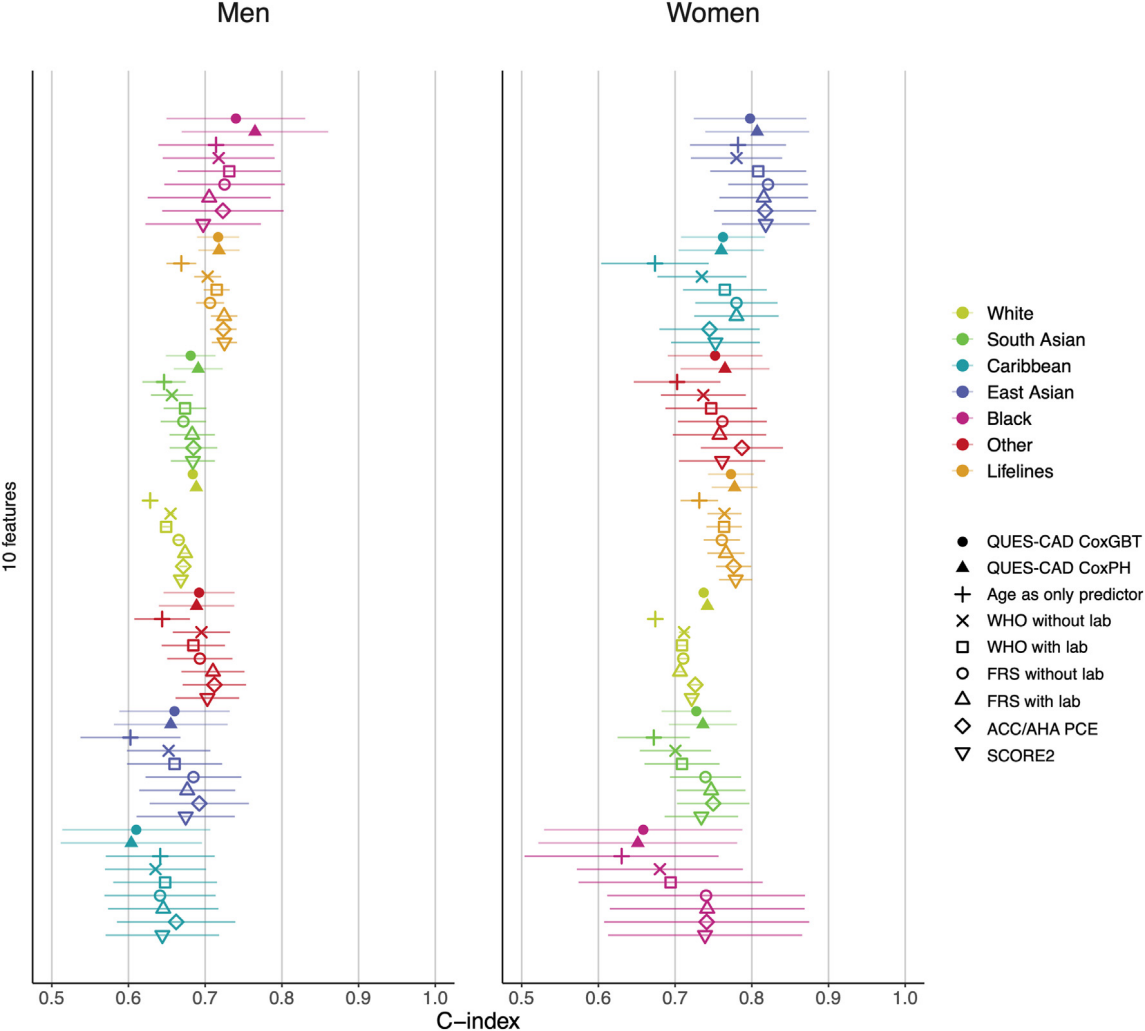
Discriminative abilities of several Coronary Artery Disease (CAD) forecasting models for men (left panel) and women (right panel). The first filled circle and triangle refer to the QUES-CAD models we developed and validated herein. Each colour-symbol combination refers to a specific model and population, explained in detail in the lateral panel. The C-index and 95% CI are presented for all models as horizontal lines. Abbreviations: CoxPH, Cox proportional hazards; CoxGBT, Cox gradient boosting; QUES-CAD, Questionnaire-Based Evaluation for Estimating Coronary Artery Disease; FRS, Framingham Coronary Heart Disease Risk Score; ACC/AHA PCE, American College of Cardiology/American Heart Association pooled cohort equation; WHO, World Health Organization; SCORE2, Systematic Coronary Risk Estimation 2.

The discriminative abilities of the QUES-CAD models and other clinical risk assessment scores in women and men across multiple ethnicities are illustrated in Fig. 2, Table 3, Table 4, Supplementary Table S7. In the models employing only questionnaire-based features, the three most significant contributing features for women were age, usual walking pace, and pack-years of smoking (Supplementary Fig. S2A and S2B), and for men, age, aspirin use, and body mass index (Supplementary Fig. S2C and S2D). Concerning the discriminative ability of QUES-CAD, for the CoxGBT models in women, the C-index varied between 0.737 for the White population and 0.782 for the “Other” ethnic population. The CoxPH models demonstrated similar C-indices ranging from 0.7 in the Black population to 0.804 in the East Asian ethnic population. In men, CoxGBT models showed inferior performance accuracy compared to what we observed among females, with a C-index ranging from 0.653 in the East Asian population to 0.737 in the Black population (Supplementary Table S7). Similar performances were demonstrated with CoxPH C-index ranging from 0.697 in the Black population to 0.808 in the East Asian population for women and in men between 0.654 in the East Asian population and 0.761 in the Black population (Supplementary Table S7). In all instances, in the external validation set, Lifelines, we observed high performances, with a C-index ranging from 0.692 to 0.771 (Supplementary Table S7). For both men and women, the QUES-CAD models (CoxGBT and CoxPH) demonstrate comparable C-index values across ethnic groups and Lifelines and have a similar discriminative capacity (based on C-index) when compared to established clinical models, i.e., ACC/AHA PCE, WHO (with and without laboratory data), FRS (with and without laboratory data), and SCORE2 (Fig. 2).

**Table 3:** Summary of significant results (based on the significant Bonferroni-adjusted p-values), as presented in Supplementary Table S9A–D, across all C-index comparisons between QUES-CAD vs established clinical risk tools.

	C-index women CoxPH	C-index men CoxPH	C-index women CoxGBT	C-index men CoxGBT
QUEST-CAD vs SCORE2	1/1	1/1	1/1	1/1
QUEST-CAD vs ACC/AHA PCE	1/1	1/1	0/0	1/1
QUEST-CAD vs FRS with lab	1/1	1/1	1/1	0/0
QUEST-CAD vs FRS without lab	1/1	1/1	1/1	1/1
QUEST-CAD vs WHO with lab	1/1	1/1	1/1	1/1
QUEST-CAD vs WHO without lab	1/1	2/2	1/1	1/1
QUEST-CAD vs Age as standalone marker	3/3	2/2	3/3	3/3

Abbreviations: PLR, partial log-likelihood ratio; SCORE2, Systematic Coronary Risk Estimation 2; WHO, World Health Organization; FRS, Framingham Coronary Heart Disease Risk Score; QUES-CAD, Questionnaire-Based Evaluation for Estimating Coronary Artery Disease; ACC/AHA, American College of Cardiology/American Heart Association; CAD, coronary artery disease; CI, confidence interval; CoxPH, cox proportional hazards; CoxGBT, cox gradient boosting.

This table illustrates the number of comparisons with statistically significant differences and shows how many of them resulted in QUES-CAD yielding significantly better outcomes. Specifically, each QUES-CAD vs existing model comparison was done in 6 ethnic subgroups of the UK Biobank and in the entire Lifelines dataset. The second number (after “/”) in the table rows shows how many of these 7 results were significant (Bonferroni adjusted), and the first number (before “/”) shows how many of these 7 results were significant in favour of QUES-CAD. All 196 comparisons are presented in greater detail in Supplementary Table S9A–D.

**Table 4:** Summary of significant results (based on the significant Bonferroni-adjusted p-values), as presented in Supplementary Table S9A–D, across all PLR comparisons between QUES-CAD vs established clinical risk tools.

	PLR women CoxPH	PLR men CoxPH	PLR women CoxGBT	PLR men CoxGBT
QUEST-CAD vs SCORE2	0/1	0/0	2/2	1/1
QUEST-CAD vs ACC/AHA PCE	0/1	0/0	2/2	1/1
QUEST-CAD vs FRS with lab	0/0	0/0	1/1	1/1
QUEST-CAD vs FRS without lab	0/0	0/0	2/2	1/1
QUEST-CAD vs WHO with lab	0/0	1/1	2/2	1/1
QUEST-CAD vs WHO without lab	0/0	1/1	2/2	2/2
QUEST-CAD vs Age as standalone marker	0/0	1/1	2/2	1/1

Abbreviations: PLR, partial log-likelihood ratio; SCORE2, Systematic Coronary Risk Estimation 2; WHO, World Health Organization; FRS, Framingham Coronary Heart Disease Risk Score; QUES-CAD, Questionnaire-Based Evaluation for Estimating Coronary Artery Disease; ACC/AHA, American College of Cardiology/American Heart Association; CAD, coronary artery disease; CI, confidence interval; CoxPH, cox proportional hazards; CoxGBT, cox gradient boosting.

This table illustrates the number of comparisons with statistically significant differences and shows how many of them resulted in QUES-CAD yielding significantly better outcomes. Specifically, each QUES-CAD vs existing model comparison was done in 6 ethnic subgroups of the UK Biobank and in the entire Lifelines dataset. The second number (after “/”) in the table rows shows how many of these 7 results were significant (Bonferroni adjusted), and the first number (before “/”) shows how many of these 7 results were significant in favour of QUES-CAD. All 196 comparisons are presented in greater detail in Supplementary Table S9A–D.

Regarding PPV and NPV, QUES-CAD models yielded very high NPVs (≥95%) and very low PPVs (≤13%), minimising false negatives while increasing the false positive results (Supplementary Table S7). Similarly, validating SCORE2 in our study, with a threshold of 7.5%, appears to return almost identical PPVs and NPVs compared to QUES-CAD across all ethnic groups and Lifelines (Supplementary Table S8).

The addition of measurement-based variables and variables that require prior medical examination (i.e., insulin use, number of treatments/medications taken, prevalent cardiovascular disease [other than CAD, and stroke diagnosed by a doctor]) and the further addition of biomarkers/other measurements (i.e., high-density lipoprotein [HDL] cholesterol, total cholesterol, and glycated haemoglobin) did not significantly improve QUES-CAD's performance in all instances, except for the White population (Supplementary Fig. S3, Supplementary Tables S10 and S11). The feature importance and hazard ratios for variables included in the models with questionnaire & measurement-based variables (or variables that require prior medical examination)-based as well as the ones including biomarkers/other measurements, are presented in Supplementary Figs. S4A–D and S5A–D.

### Risk stratification of QUES-CAD is comparable to established clinical models

Subsequently, we performed a comparison between the performance of the easier-to-use QUES-CAD model and the comparator-established clinical models determining both the discriminatory power (C-index) and goodness-of-fit (PLR). Our results show that the QUES-CAD model achieved similar C-index and PLR values (not statistically different based on the external Lifelines cohort) to those of clinical models across multiple populations. Specifically, in the training set (using 10-fold cross-validation), White males and females, QUES-CAD CoxGBT showed overall significantly higher C-indices and PLRs (Bonferroni-adjusted significance thresholds) compared to all comparator clinical models (Table 3, Table 4, Supplementary Table S9A–D). For the rest of the UK Biobank ethnicities no significant differences were evident for either C-index or PLR (Table 3, Table 4, Supplementary Table S9A–D). Importantly, CoxGBT performed better than CoxPH, in terms of fitting the data (Table 3, Table 4, Supplementary Table S9A–D). The only instances where the comparator models appear to fit the data of the external validation set, Lifelines, better (significant Bonferroni-adjusted p-value for PLR comparisons) is when comparing SCORE2 and ACC/AHA PCE to CoxPH in women (Supplementary Table S9D); although, for the same comparisons with CoxGBT, QUES-CAD fit the data significantly better than both SCORE2 and ACC/AHA PCE (Table 4, Supplementary Table S9B).

**Fig. 3:**
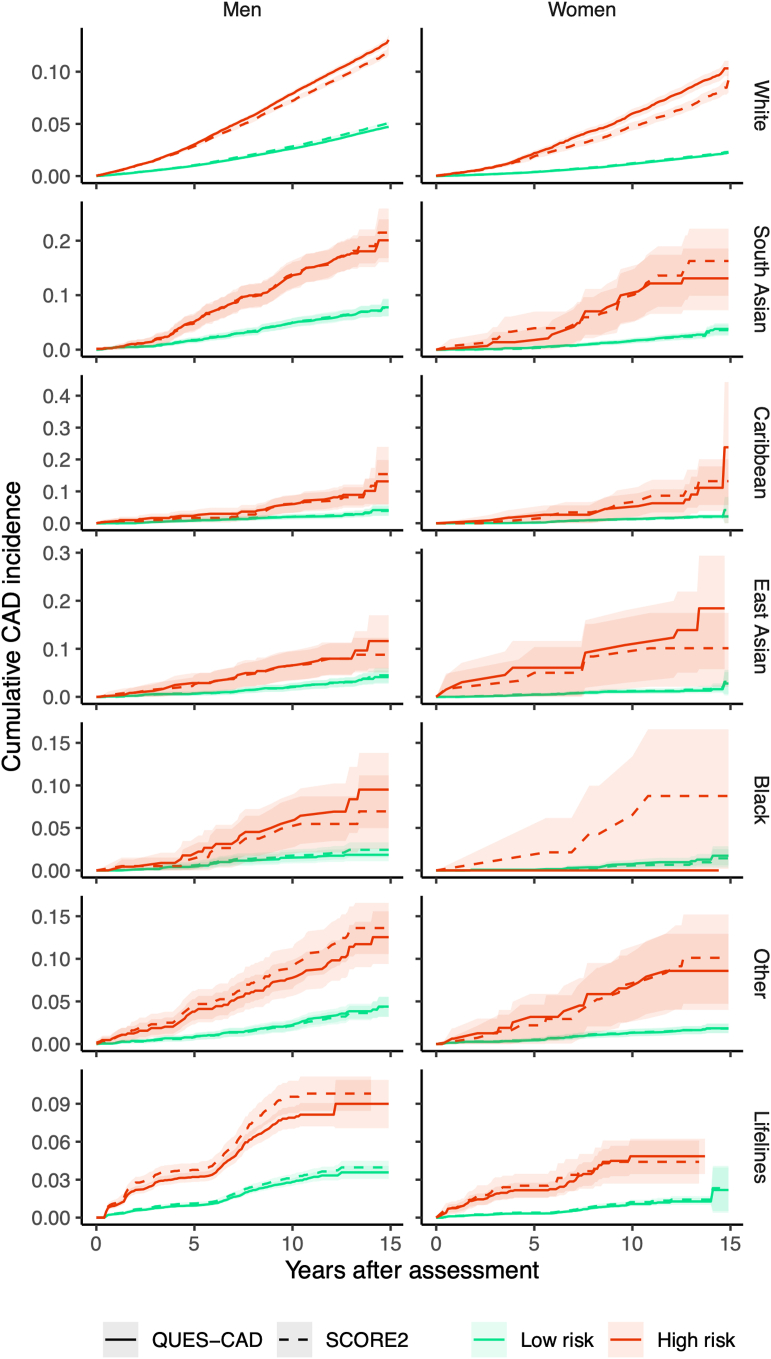
Cumulative incidence of CAD by ethnicity and sex over time. The x-axis represents years after baseline (initial assessment), while the y-axis indicates the cumulative CAD incidence. Data are stratified by sex (men and women) and population, including White, South Asian, Caribbean, East Asian, Black, Other, and Lifelines (external validation cohort). Cumulative incidence curves are plotted for low and high-risk groups according to QUES-CAD CoxGBT and SCORE2 thresholds; for QUES-CAD we used the threshold that returned the same group size as SCORE2 in the White population. The lighter-coloured lines represent the 95% CI. Abbreviations: CAD, coronary artery disease; QUES-CAD, Questionnaire-Based Evaluation for Estimating Coronary Artery Disease; CoxGBT, Cox gradient boosting; SCORE2, Systematic Coronary Risk Estimation 2; CI, confidence interval.

Regarding the 15-year CAD risk stratification, comparing QUES-CAD to the widely validated SCORE2 while maintaining the same high-risk group size for the White population, both QUES-CAD and SCORE2 perform comparably in all populations, and both high-risk groups (of QUES-CAD and SCORE2) have an almost identical incident risk, without significant differences (Fig. 3, Supplementary Fig. S6). Similarly, low-risk groups also have an identical CAD incidence risk across all populations (Fig. 3, Supplementary Fig. S6).

## Discussion

In this study including 574,021 individuals, we sought to develop and evaluate the performance of our novel questionnaire-based non-laboratory risk stratification model for incident CAD. Furthermore, we internally validated QUES-CAD in ethnic minorities that are more prevalent than in other European countries; yet they approximate the predicted ethnic make-up of other European countries by 2030.^24^ Additionally, we externally validated the developed models in Lifelines, one of the largest European biobanks, to examine the generalisability of our results. The primary finding was that the QUES-CAD model stratifies incident CAD as accurately as current clinical models such as FRS, SCORE2, ACC/AHA PCE, and WHO in all populations and also achieved relatively higher performance in the external validation cohort, suggesting its potential applicability for the multi-ethnic populations of Europe. The inclusion of physical measurements and/or biomarkers (variables presented in Table 1, Table 2, Supplementary Figs. S4A–D and S5A–D) did not increase the performance of QUES-CAD. At the same time, with the currently available tools and the present limitations of healthcare systems, creating a population risk stratification program that requires laboratory values for CAD may comprise an unrealistic goal. This is mainly due to the considerable costs and logistical challenges associated with the need for blood sample collection, but also for the organisation and management of “screening units” to perform these tests.^32^ Specifically, as described in the 2018 European Commission report regarding inequalities in healthcare access, attracting and retaining health professionals is problematic, and reaching particularly vulnerable communities with limited access to qualitative healthcare is burdensome.^20^ These results suggest that a questionnaire-based risk stratification algorithm performs at least as well as currently available tools (comprising a comparable number of features) that employ physical and blood biomarkers, enable new scalable avenues for risk screening with major implications for population health monitoring. A questionnaire-based model can be applied population-wide due to the limited cost and effort, as laboratory tests or time from medical personnel are no longer required. Notably, the specific variables selected to construct the QUES-CAD model exclude any variables that individuals find difficult to assess accurately, such as waist circumference or body fat percentage. Instead, only those questionnaire variables that were asked directly to the biobanks’ participants were included, ensuring that these answers translate one-on-one to the answers that can be expected in a population-wide screening. This finding implies that these models are uniquely suited to be deployed without requiring assistance from medical personnel.

QUES-CAD may also enhance disease prevention in resource-limited settings where access to preventive cardiovascular care is limited.^29^ With the primary goal to improve health equity and reduce health disparities while reducing the burden of CAD as a highly prevalent non-communicable disease (NCD), QUES-CAD is a readily scalable solution to accompany or replace the currently implemented risk models, especially in low socioeconomic status (SES) or resource-limited settings, i.e., in rural European areas. The Lancet NCD Action Group and the NCD Alliance suggested that cardiovascular risk reduction ranks among the five top priority interventions for NCDs.^33^ Notably, in the same study, tobacco use ranks first, while the category “obesity, unhealthy diet, and physical inactivity” ranked third.^33^ Therefore, a questionnaire-based risk stratification tool comprising such features, along with household income information, provides a solution to stratify individuals at a population level to facilitate the effective deployment of these interventions.

Despite growing efforts towards advancing ML initiatives in cardiovascular care, most of these applications focus on imaging, electrocardiography, and biomarker analyses.^34^ The development of QUES-CAD allows the novelty of reliable non-laboratory stratification methods to be implemented in the most recent and ever-increasing technological trends in clinical decision-making, such as the emerging use of large language models (LLM) for telemedicine applications (remote patient monitoring). LLMs can provide a suitable avenue to integrate QUES-CAD, making it user-friendly and widely accessible for virtual care.^35^^,^^36^ For instance, users can provide input to a chatbot based on the questions included in QUES-CAD, and the chatbot can predict their ten-year risk of developing CAD. Furthermore, it is pertinent to mention that LLMs can receive both structured and unstructured data from the patient directly or from electronic health records, being able to adjust their prediction based on the inputs it receives across the patient's lifetime.^37^ Moreover, QUES-CAD showed greater uniformity in its discriminative performance across ethnicities compared to currently used clinical tools, and the absence of blood biomarkers enables individual risk calculation outside the “strict” healthcare setting, such as in a remote or hybrid environment. Even at primary care visits, QUES-CAD can be performed during consultation hours with the general practitioner via a website or mobile app and readily provides the individual risk for developing CAD over the next 15 years with ten simple-to-obtain questions, almost all of which are typically asked during a patient visit at a medical facility.

Since CoxGBT can capture non-linear relationships, our results suggest that the association between the covariates and the hazard is not entirely linear. Although CoxGBT performs better than the CoxPH model in Lifelines in terms of goodness-of-fit, similar results could potentially be achieved with a CoxPH model by incorporating interaction terms or non-linear transformations, such as quadratic terms. Given that GBT models are often regarded as “black box” models, it would be advantageous to focus on optimising the regression (CoxPH) models, as their coefficients provide greater interpretability due to their linear structure. Examining the decision trees from the GBT model could also help uncover key interaction effects or inform necessary variable transformations, offering insights that might improve the regression models.

At the same time, we aim to bridge the sex gap by generating separate models for males and females that are optimised to yield the highest performance for each sex. Interestingly, the female version of QUES-CAD demonstrates higher C-indices, and besides this finding being also reported in other risk tools, different models (with discrete variables) for men and women are currently absent in clinical practice.

The current study presents several strengths and limitations. First, this study achieves the highest validation standards in the ML field by showing the ML-based models' performance and potential clinical utility of a questionnaire-based risk stratification model for incident CAD in two large population cohorts across multiple ethnicities. From a modelling perspective, this minimises the chances of overfitting and provides evidence of the model's validity. Then, we further underpinned the reliability of our models by validating them in all ethnic populations of the UK Biobank and provided a comparison of the six major clinical risk stratification tools: FRS without lab, FRS with lab, ACC/AHA PCE, WHO without lab, WHO with lab, and SCORE2. One limitation is that ethnicity data may only be partially accurate, as with all self-reported biobank data. In particular, an individual's self-reported ethnicity may be shaped by their perceptions and cultural and societal influences and may not consistently be representative of their ancestral background. However, these biases are in some way desirable when the aim is to deploy the QUES-CAD on a population-wide scale, as in this case, these biases will be part of the assessment and accounted for by the models. Additionally, daily aspirin use (as a predictor) may be a marker of a clinical encounter and increased CVD risk. Lastly, since this study is observational, it is not possible to establish cause-and-effect relationships between the variables integrated into QUES-CAD and the anticipated outcomes.

### Conclusion

In conclusion, QUES-CAD, a novel ML-based multi-ethnic CAD incident risk stratification tool, solely employs ten questionnaire-based variables and performs comparable to the established risk scoring systems (which require lab-based variables and other physical measurements) currently implemented in primary care cardiology guidelines. These questionnaire-based models reduce effort and cost to a minimum and can thereby revolutionise the monitoring of CAD risk, enabling population-wide screening to identify which individuals would benefit from preventive interventions, including both lifestyle and medical interventions, that target cardiometabolic risk in a cost-effective and scalable manner.

## Contributors

MK contributed to the conceptualisation, data curation, validation, investigation, methodology, project administration, visualisation, manuscript writing, and manuscript review. PF contributed to the data analysis, data curation, investigation, methodology, validation, and visualisation. MK, PF, and SvD accessed and verified the underlying data. FA contributed to the visualisation, manuscript writing, and manuscript review. NS, MA, RS, RHH, HP, JJB, and DEA contributed to the manuscript review. BHRW contributed to the data curation, manuscript review, resources, and funding acquisition. JCF contributed to the conceptualisation, methodology, manuscript writing, and manuscript review. CSM and SvD contributed equally to the conceptualisation, data curation, validation, methodology, project administration, visualisation, manuscript writing, manuscript review, and supervision. All authors read and approved the final version of the manuscript.

## Data sharing statement

Study data are available from UK Biobank and Lifelines but were used under licence for the current study, which restricts their public availability. Data may be obtained from a third party and are not publicly available. Researchers can apply to use the UK Biobank and Lifelines data used in this study. More information about how to request UK Biobank data and the conditions of use can be found on their website (https://www.ukbiobank.ac.uk/enable-your-research/apply-for-access), and for Lifelines data, and the conditions of use can be found on their website (https://www.lifelines-biobank.com/researchers/working-with-us). The underlying code is available and can be requested from the corresponding author.

## Declaration of interests

PF, SvD, and JCF are employed by Ancora Health B.V. and own shares of Ancora Health B.V. BHRW sits on the medical advisory board of Ancora Health B.V., without being compensated for this position. All other authors have no conflict of interest to declare.

## Figures and Tables

**Figure:**