# Supplementary material for: Simplifying coronary artery disease risk stratification: development and validation of a questionnaire-based alternative comparable to clinical risk tools
Source: eBioMedicine. 2024 Dec 25;111:105518. doi: 10.1016/j.ebiom.2024.105518 (PMC11732493; doi:10.1016/j.ebiom.2024.105518)
Supplement: Supplementary Material [file mmc1.docx]

**Supplementary Material**

**Table of Contents**

[Supplementary methods data 3](#_Toc183123805)

[Supplementary Figure S1A. Follow-up time for the UK Biobank until October 2022. 3](#_Toc183123806)

[Supplementary Figure S1B. Follow-up time for Lifelines. 3](#_Toc183123807)

[Supplementary Table S1A. UK Biobank Coronary Artery Disease annotation. 4](#_Toc183123808)

[Supplementary Table S1B. Lifelines Coronary Artery Disease annotation. 4](#_Toc183123809)

[Supplementary Table S2. Mapped features between the UK Biobank and Lifelines. 5](#_Toc183123810)

[Supplementary Table S3. Ethnic population formulations in the UK Biobank. 6](#_Toc183123811)

[Supplementary Table S4. Parameters used for training the gradient boosted survival models. 7](#_Toc183123812)

[Supplementary Table S5. Calculation of time-dependent positive predictive value (PPV) and negative predictive value (NPV) over ten years. 7](#_Toc183123813)

[Supplementary Table S6. Ethnic makeup of the Lifelines cohort as established at baseline. 8](#_Toc183123814)

[QUES-CAD (Questionnaire-only) 9](#_Toc183123815)

[Supplementary Figure S2A. Questionnaire-based feature importance for coronary artery disease in women. 9](#_Toc183123816)

[Supplementary Figure S2B. Questionnaire-based features’ hazard ratios for coronary artery disease prediction in women. 10](#_Toc183123817)

[Supplementary Figure S2C. Questionnaire-based feature importance with coronary artery disease prediction in men. 11](#_Toc183123818)

[Supplementary Figure S2D. Questionnaire-based features’ hazard ratios for coronary artery disease prediction in men. 12](#_Toc183123819)

[Supplementary Table S7. Diagnostic metrics of the developed models containing questionnaire-only features. 12](#_Toc183123820)

[Supplementary Table S8. Diagnostic metrics of SCORE2. 14](#_Toc183123821)

[Supplementary Table S9A. Comparison between the C-indices, PL, and PLR of QUES-CAD and established clinical prediction algorithms and age as a standalone variable across six ethnicities and an external validation cohort using CoxGBT in male subjects. 15](#_Toc183123822)

[Supplementary Table S9B. Comparison between the C-indices, PL, and PLR of QUES-CAD and established clinical prediction algorithms and age as a standalone variable across six ethnicities and an external validation cohort using CoxGBT in women. 18](#_Toc183123823)

[Supplementary Table S9C. Comparison between the C-indices, PL, and PLR of QUES-CAD and established clinical prediction algorithms and age as a standalone variable across six ethnicities and an external validation cohort using CoxPH in male subjects. 20](#_Toc183123824)

[Supplementary Table S9D. Comparison between the C-indices, PL, and PLR of QUES-CAD and established clinical prediction algorithms and age as a standalone variable across six ethnicities and an external validation cohort using CoxPH in women. 23](#_Toc183123825)

[Supplementary Figure S3. Predictive abilities of the several Coronary Artery Disease forecasting models constructed in this study for men (left panel) and women (right panel). 26](#_Toc183123826)

[Questionnaire & Measurement-based variables (or variables that require prior medical examination) 28](#_Toc183123827)

[Supplementary Figure S4A. Questionnaire & Measurement-based variables (or variables that require prior medical examination)-based feature importance with coronary artery disease prediction in women. 28](#_Toc183123828)

[Supplementary Figure S4B. Questionnaire & Measurement-based variables (or variables that require prior medical examination)-based features’ hazard ratios for coronary artery disease prediction in women. 29](#_Toc183123829)

[Supplementary Figure S4C. Questionnaire & Measurement-based variables (or variables that require prior medical examination)-based feature importance with coronary artery disease prediction in men. 30](#_Toc183123830)

[Supplementary Figure S4D. Questionnaire & Measurement-based variables (or variables that require prior medical examination)-based features’ hazard ratios for coronary artery disease prediction in men. 31](#_Toc183123831)

[Supplementary Table S10. Diagnostic metrics of the developed models containing questionnaire & measurement-based variables (or variables that require prior medical examination) features. 31](#_Toc183123832)

[Questionnaire, measurement-based variables (or variables that require prior medical examination), and biomarkers 34](#_Toc183123833)

[Supplementary Figure S5A. Questionnaire, measurement-based variables (or variables that require prior medical examination), and biomarkers-based feature importance with coronary artery disease in women. 34](#_Toc183123834)

[Supplementary Figure S5B. Questionnaire, measurement-based variables (or variables that require prior medical examination), and biomarkers-based features’ hazard ratios for coronary artery disease prediction in women. 35](#_Toc183123835)

[Supplementary Figure S5C. Questionnaire, measurement-based variables (or variables that require prior medical examination), and biomarkers-based feature importance with coronary artery disease in men. 36](#_Toc183123836)

[Supplementary Figure S5D. Questionnaire, measurement-based variables (or variables that require prior medical examination), and biomarkers-based features’ hazard ratios for coronary artery disease prediction in men. 37](#_Toc183123837)

[Supplementary Table S11. Diagnostic metrics of the developed models containing questionnaire, measurement-based variables (or variables that require prior medical examination), and biomarkers. 37](#_Toc183123838)

[Risk stratification 40](#_Toc183123839)

[Supplementary Figure S6. Cumulative incidence of coronary artery disease by ethnicity and sex over time. 40](#_Toc183123840)

# Supplementary methods data

## Supplementary Figure S1A. Follow-up time for the UK Biobank until October 2022.

## Supplementary Figure S1B. Follow-up time for Lifelines.

## Supplementary Table S1A. UK Biobank Coronary Artery Disease annotation.

| **Diagnosis** | | **Age of diagnosis** | |
| --- | --- | --- | --- |
| **Data-Field** | **Name** | **Data-Field** | **Name** |
| 20002 | Non-cancer illness code, self-reported | 20009 | Interpolated Age of participant when non-cancer illness first diagnosed |
| 20004 | Operation-code | 20011 | Interpolated Age of participant when operation took place |
| 6150 | Vascular/heart problems diagnosed by doctor | 3894 | Age heart attack diagnosed |
| 41271 | Diagnoses – ICD9 | 41281 | Date of first in-patient diagnosis - ICD9 |
| 41270 | Diagnoses - ICD10 | 41280 | Date of first in-patient diagnosis - ICD10 |
| 41272 | Operative procedures - OPCS4 | 41282 | Date of first operative procedure - OPCS4 |

## Supplementary Table S1B. Lifelines Coronary Artery Disease annotation.

| **Prevalent diagnosis** | | **Incident diagnosis** | |
| --- | --- | --- | --- |
| **Variable** | **Age of diagnosis** | **Variable** | **Age of diagnosis** |
| heartattack_presence_adu_q_1 | heartattack_startage_adu_q_1 | heartattack_followup_adu_q_1 | Manually calculated by mean (age at assessment when diagnosis reported, age previous assessment). |
| heartattack_presence_adu_q_2 | heartattack_presence_adu_q_2_a | angioplasty_bypass_adu_q_1 |  |
| angioplasty_bypass_adu_q_1 | angioplasty_bypass_adu_q_1_a | angioplasty_followup_adu_q_1 |  |
| other_disease_adu_q_1_* == “ANGIOPLAS\|CBAG\|DOTTER\|HARTAANVAL\|HARTINFARCT\|HARTOPERATIE.*(PASS\|OMLEIDING)” |  | otherdisease_followup_adu_q_1_* == ANGIOPLAS\|CBAG\|DOTTER\|HARTAANVAL\|HARTINFARCT\|HARTOPERATIE.*(PASS\|OMLEIDING) |  |

## Supplementary Table S2. Mapped features between the UK Biobank and Lifelines.

For ordinal UK Biobank features, numeric data values were used. Subsequently, each category was one hot encoded; these feature names are depicted by “category -- UK Biobank feature_0.0”.

| **Feature UK Biobank** | **Feature Lifelines** | **Notes** |
| --- | --- | --- |
| Age of attending assessment centre_0.0 | age.1a | In the UK Biobank, more specific ages of attending the assessment centre were calculated based on the date of attending the assessment centre minus the birth year/month of the participant. |
| Alcohol intake frequency._0.0 | ffqh_alcohol_adu_q_27 | Alcohol intake categories did not match exactly between UK Biobank and Lifelines. The Lifelines categories were therefore mapped as closely as possible to the UK Biobank categories. |
| Aspirin -- Medication for pain relief, constipation, heartburn_0.0 | otc_painfever_adu_q_1.1a |  |
| Average total household income before tax LL mapped_0.0 | income_net_adu_q_1_v2.1a | "The UK Biobank income is before tax while the Lifelines income is after tax. The categories were therefore mapped as followed: |
| Category mapping  UK Biobank 1 (Less than £18,000) = Lifelines 9, 10, 11 (less than €1500)  UK Biobank 2 (£18,000 – £30,999) = Lifelines 12, 13 (€1500 - €2500)  UK Biobank 3 (£31,000 – £51,999) = Lifelines 14 (€2500 - €3000)  UK Biobank 4, 5 (Greater than £51,999) = Lifelines 15, 16 (Greater than €3000) | | |
| Body mass index (BMI)_0.0 | bodyweight_kg_all_m_1.1a / (bodylength_cm_all_m_1.1a / 100)^2 |  |
| Cholesterol_0.0 | cholesterol_result_all_m_1.1a |  |
| Current -- Smoking status_0.0 | smoking_current_adu_q_1.1a |  |
| CVD__ICD9_ICD10_OPSC4_selfreported_doctor.before_first_assessment | heartattack_presence_adu_q_1.1a, aneurysm_diagnosis_adu_q_1.1a, angioplasty_bypass_adu_q_1.1a, arrhythmia_diagnosis_adu_q_1.1a, arrhythmia_presence_adu_q_2.1a, heartfailure_presence_adu_q_1.1a, heartfailure_presence_adu_q_2.1a, stroke_presence_adu_q_1.1a, stroke_presence_adu_q_2.1a, carotid_stenosis_adu_q_1.1a, heartvalve_presence_adu_q_1.1a, atherosclerosis_presence_adu_q_1.1a, heartattack_presence_adu_q_2.1a, aneurysm_presence_adu_q_2.1a, stenosis_presence_adu_q_1.1a, cvd_followup_adu_q_1 .1a, heartattack_followup_adu_q_1.1a, stroke_followup_adu_q_1.1a, heartfailure_followup_adu_q_1.1a, claudication_followup_adu_q_1.1a, angioplasty_followup_adu_q_1.1a, thrombosis_presence_adu_q_1.1a, embolism_presence_adu_q_1.1a |  |
| Glycated haemoglobin (HbA1c)_0.0 | hba1cconc_result_all_m_1.1a |  |
| HDL cholesterol_0.0 | hdlchol_result_all_m_1.1a |  |
| Heart disease -- Illnesses of father_0.0 | cvd_father_fam_q_1_a.1a, cvd_father_fam_q_1_b.1a |  |
| Heart disease -- Illnesses of mother_0.0 | cvd_mother_fam_q_1_a.1a, cvd_mother_fam_q_1_b.1a |  |
| Number of cigarettes currently smoked daily (current cigarette smokers)_0.0 | cigarettes_frequency_adu_q_1.1a |  |

## Supplementary Table S3. Ethnic population formulations in the UK Biobank.

| **Ethnic population** | **UK Biobank entries (Data-Field 21000: Ethnic background)** |
| --- | --- |
| White | White, British, Irish, Any other white background |
| South Asian | Indian, Pakistani, Bangladeshi |
| Caribbean | Caribbean, White and Black Caribbean |
| East Asian | Asian or Asian British, Chinese, White and Asian, Any other Asian background |
| Black | Black or Black British, African, White and Black African, Any other Black background |
| Other | Other ethnic population, Do not know, Prefer not to answer, Mixed, Any other mixed background |

## Supplementary Table S4. Parameters used for training the gradient boosted survival models.

| **Parameter** | **Value** |
| --- | --- |
| n_estimators | 100 |
| learning_rate | 0.1 |
| max_depth | 3 |
| validation_fraction | 0.15 |
| n_iter_no_change | 5 |
| min_samples_split | 10 |
| sample weights | 1 for controls, N controls/N cases for cases |

## Supplementary Table S5. Calculation of time-dependent positive predictive value (PPV) and negative predictive value (NPV) over ten years.

| **Value** | **Calculation** |
| --- | --- |
| PPV | (sensitivity * incidence) / (sensitivity * incidence + (1 - specificity) * (1 - incidence)) |
| NPV | (specificity * (1 - incidence)) / (specificity * (1 - incidence) + (1 - sensitivity) * incidence) |

## Supplementary Table S6. Ethnic makeup of the Lifelines cohort as established at baseline.

| **Ethnic population** | **Frequency** | **Percentage** |
| --- | --- | --- |
| Asian | 83 | 0.4 |
| Black | 29 | 0.1 |
| NA | 1207 | 5.9 |
| Other | 142 | 0.7 |
| White/eastern and western European | 18975 | 92.6 |
| White/Mediterranean or Arabic | 46 | 0.2 |

# QUES-CAD (Questionnaire-only)

## Supplementary Figure S2A. Questionnaire-based feature importance for coronary artery disease in women.

## Supplementary Figure S2B. Questionnaire-based features’ hazard ratios for coronary artery disease prediction in women.

## Supplementary Figure S2C. Questionnaire-based feature importance with coronary artery disease prediction in men.

## Supplementary Figure S2D. Questionnaire-based features’ hazard ratios for coronary artery disease prediction in men.

## Supplementary Table S7. Diagnostic metrics of the developed models containing questionnaire-only features.

| **Sex** | **Model** | **Ethnicity** | **C-index** | **N** | **Low risk** | **High risk** | **Sensitivity (95% CI)** | **Specificity (95% CI)** | **PPV (95% CI)** | **NPV (95% CI)** | **Threshold** | **10-year incidence** |
| --- | --- | --- | --- | --- | --- | --- | --- | --- | --- | --- | --- | --- |
| men | CoxPH | White | 0.689 (0.684-0.693) | 177301 | 130152 | 47149 | 53 (52-55) | 74 (74-75) | 8 (7-8) | 98 (98-98) | 0.046 | 0.037 |
| men | CoxPH | South Asian | 0.694 (0.666-0.721) | 3411 | 2509 | 902 | 51 (45-59) | 75 (74-77) | 13 (11-15) | 96 (95-96) | 0.046 | 0.066 |
| men | CoxPH | Caribbean | 0.678 (0.609-0.747) | 1538 | 1217 | 321 | 44 (27-57) | 80 (78-81) | 6 (3-8) | 98 (98-99) | 0.046 | 0.026 |
| men | CoxPH | East Asian | 0.654 (0.595-0.714) | 1572 | 1271 | 301 | 37 (22-53) | 81 (79-83) | 5 (3-8) | 98 (97-98) | 0.046 | 0.026 |
| men | CoxPH | Black | 0.761 (0.683-0.839) | 1577 | 1329 | 248 | 51 (32-67) | 85 (83-86) | 6 (4-9) | 99 (98-99) | 0.046 | 0.019 |
| men | CoxPH | Other | 0.685 (0.647-0.724) | 2779 | 2089 | 690 | 58 (48-70) | 76 (74-78) | 8 (6-10) | 98 (98-99) | 0.046 | 0.033 |
| men | CoxPH | Lifelines | 0.692 (0.673-0.71) | 39651 | 29887 | 9764 | 46 (43-50) | 76 (76-77) | 7 (7-8) | 97 (97-97) | 0.046 | 0.04 |
| men | CoxGBT | White | 0.684 (0.679-0.689) | 177301 | 130152 | 47149 | 53 (52-54) | 74 (74-75) | 7 (7-8) | 98 (98-98) | 0.276 | 0.037 |
| men | CoxGBT | South Asian | 0.68 (0.652-0.708) | 3411 | 2607 | 804 | 47 (40-52) | 78 (77-79) | 13 (11-15) | 95 (95-96) | 0.276 | 0.066 |
| men | CoxGBT | Caribbean | 0.677 (0.604-0.751) | 1538 | 1216 | 322 | 43 (31-63) | 80 (78-81) | 5 (4-8) | 98 (98-99) | 0.276 | 0.026 |
| men | CoxGBT | East Asian | 0.653 (0.595-0.711) | 1572 | 1318 | 254 | 37 (18-48) | 84 (82-86) | 6 (3-9) | 98 (97-98) | 0.276 | 0.026 |
| men | CoxGBT | Black | 0.737 (0.664-0.811) | 1577 | 1333 | 244 | 41 (24-61) | 85 (83-87) | 5 (3-8) | 99 (98-99) | 0.276 | 0.019 |
| men | CoxGBT | Other | 0.685 (0.649-0.721) | 2779 | 2095 | 684 | 54 (44-65) | 76 (75-78) | 7 (6-9) | 98 (98-98) | 0.276 | 0.033 |
| men | CoxGBT | Lifelines | 0.699 (0.681-0.717) | 39651 | 30529 | 9122 | 46 (42-49) | 78 (77-78) | 8 (7-9) | 97 (97-97) | 0.276 | 0.04 |
| women | CoxPH | White | 0.743 (0.736-0.749) | 214108 | 201214 | 12894 | 25 (24-27) | 94 (94-94) | 6 (5-6) | 99 (99-99) | 0.036 | 0.014 |
| women | CoxPH | South Asian | 0.726 (0.682-0.769) | 2977 | 2806 | 171 | 24 (13-35) | 95 (94-95) | 8 (4-14) | 98 (98-99) | 0.036 | 0.02 |
| women | CoxPH | Caribbean | 0.76 (0.704-0.815) | 2645 | 2534 | 111 | 15 (4-25) | 96 (95-97) | 5 (1-9) | 99 (99-99) | 0.036 | 0.013 |
| women | CoxPH | East Asian | 0.808 (0.743-0.873) | 1901 | 1835 | 66 | 29 (9-47) | 97 (96-97) | 11 (3-19) | 99 (99-99) | 0.036 | 0.013 |
| women | CoxPH | Black | 0.697 (0.581-0.812) | 1626 | 1572 | 54 | 0 (0-0) | 97 (96-98) | 0 (0-0) | 99 (99-99) | 0.036 | 0.007 |
| women | CoxPH | Other | 0.745 (0.694-0.796) | 3320 | 3150 | 170 | 22 (12-33) | 95 (94-96) | 7 (3-11) | 99 (99-99) | 0.036 | 0.016 |
| women | CoxPH | Lifelines | 0.771 (0.748-0.794) | 55697 | 50583 | 5114 | 30 (24-35) | 91 (91-91) | 5 (4-6) | 99 (99-99) | 0.036 | 0.014 |
| women | CoxGBT | White | 0.737 (0.731-0.744) | 214108 | 201214 | 12894 | 26 (25-27) | 94 (94-94) | 6 (5-6) | 99 (99-99) | 0.379 | 0.014 |
| women | CoxGBT | South Asian | 0.718 (0.672-0.763) | 2977 | 2818 | 159 | 29 (18-40) | 95 (94-96) | 11 (6-16) | 99 (98-99) | 0.379 | 0.02 |
| women | CoxGBT | Caribbean | 0.768 (0.712-0.824) | 2645 | 2528 | 117 | 18 (4-32) | 96 (95-97) | 5 (1-11) | 99 (99-99) | 0.379 | 0.013 |
| women | CoxGBT | East Asian | 0.804 (0.732-0.875) | 1901 | 1840 | 61 | 21 (7-40) | 97 (96-98) | 8 (2-20) | 99 (99-99) | 0.379 | 0.013 |
| women | CoxGBT | Black | 0.7 (0.583-0.817) | 1626 | 1570 | 56 | 0 (0-0) | 97 (96-97) | 0 (0-0) | 99 (99-99) | 0.379 | 0.007 |
| women | CoxGBT | Other | 0.74 (0.686-0.794) | 3320 | 3142 | 178 | 24 (12-36) | 95 (94-96) | 7 (3-11) | 99 (99-99) | 0.379 | 0.016 |
| women | CoxGBT | Lifelines | 0.759 (0.736-0.783) | 55697 | 51507 | 4190 | 24 (19-29) | 93 (93-93) | 5 (4-6) | 99 (99-99) | 0.379 | 0.014 |

Abbreviations: CI, confidence interval; PPV, positive predictive value; NPV, negative predictive value; CoxPH, Cox proportional hazards; CoxGBT, Cox gradient boosting.

## Supplementary Table S8. Diagnostic metrics of SCORE2.

| **Sex** | **Model** | **Ethnicity** | **C-index** | **N** | **Low risk** | **High risk** | **Sensitivity (95% CI)** | **Specificity (95% CI)** | **PPV (95% CI)** | **NPV (95% CI)** | **Threshold** | **10-year incidence** |
| --- | --- | --- | --- | --- | --- | --- | --- | --- | --- | --- | --- | --- |
| men | SCORE2 | White | 0.668 (0.663-0.673) | 177301 | 130152 | 47149 | 49 (48-50) | 74 (74-74) | 7 (7-7) | 97 (97-97) | 0.075 | 0.037 |
| men | SCORE2 | South Asian | 0.684 (0.655-0.713) | 3411 | 2654 | 757 | 45 (38-51) | 79 (78-81) | 13 (11-16) | 95 (95-96) | 0.075 | 0.066 |
| men | SCORE2 | Caribbean | 0.644 (0.57-0.718) | 1538 | 1227 | 311 | 43 (28-59) | 80 (79-82) | 6 (3-8) | 98 (98-99) | 0.075 | 0.026 |
| men | SCORE2 | East Asian | 0.675 (0.611-0.739) | 1572 | 1293 | 279 | 42 (26-58) | 83 (81-85) | 6 (4-10) | 98 (98-99) | 0.075 | 0.026 |
| men | SCORE2 | Black | 0.697 (0.622-0.773) | 1577 | 1374 | 203 | 31 (19-52) | 87 (86-89) | 5 (3-9) | 98 (98-99) | 0.075 | 0.019 |
| men | SCORE2 | Other | 0.703 (0.661-0.744) | 2779 | 2178 | 601 | 54 (43-64) | 79 (78-81) | 8 (6-10) | 98 (98-98) | 0.075 | 0.033 |
| men | SCORE2 | Lifelines | 0.725 (0.709-0.742) | 39651 | 34295 | 5356 | 32 (29-36) | 87 (87-88) | 10 (9-11) | 97 (97-97) | 0.075 | 0.04 |
| women | SCORE2 | White | 0.722 (0.715-0.729) | 214108 | 201214 | 12894 | 20 (19-22) | 94 (94-94) | 5 (4-5) | 99 (99-99) | 0.075 | 0.014 |
| women | SCORE2 | South Asian | 0.734 (0.686-0.782) | 2977 | 2811 | 166 | 24 (13-34) | 95 (94-96) | 9 (4-13) | 98 (98-99) | 0.075 | 0.02 |
| women | SCORE2 | Caribbean | 0.753 (0.695-0.81) | 2645 | 2511 | 134 | 21 (10-39) | 95 (95-96) | 5 (2-11) | 99 (99-99) | 0.075 | 0.013 |
| women | SCORE2 | East Asian | 0.818 (0.761-0.875) | 1901 | 1833 | 68 | 21 (7-37) | 97 (96-98) | 7 (2-16) | 99 (99-99) | 0.075 | 0.013 |
| women | SCORE2 | Black | 0.739 (0.613-0.866) | 1626 | 1572 | 54 | 25 (3-58) | 97 (96-98) | 6 (1-16) | 99 (99-100) | 0.075 | 0.007 |
| women | SCORE2 | Other | 0.761 (0.705-0.817) | 3320 | 3162 | 158 | 22 (11-33) | 96 (95-96) | 7 (4-12) | 99 (99-99) | 0.075 | 0.016 |
| women | SCORE2 | Lifelines | 0.779 (0.757-0.801) | 55697 | 52803 | 2894 | 18 (13-22) | 95 (95-95) | 5 (4-6) | 99 (99-99) | 0.075 | 0.014 |

Abbreviations: CI, confidence interval; PPV, positive predictive value; NPV, negative predictive value; SCORE2, Systematic Coronary Risk Estimation 2.

## Supplementary Table S9A. Comparison between the C-indices, PL, and PLR of QUES-CAD and established clinical prediction algorithms and age as a standalone variable across six ethnicities and an external validation cohort using CoxGBT in male subjects.

| **Population** | **QUES-CAD** | **Comparator model** | **C-index QUES-CAD (95% CI)** | **C-index comparator model (95% CI)** | **C-index QUES-CAD-C-index comparator model** | **PL**  **QUES-CAD** | **PL**  **comparator model** | **PLR** | **C-index**  **Bonferroni-adjusted p-value** | **PLR**  **Bonferroni-adjusted p-value** |
| --- | --- | --- | --- | --- | --- | --- | --- | --- | --- | --- |
| White | QUES-CAD | SCORE2 | 0.682 (0.677-0.688) | 0.669 (0.663-0.674) | 0.013 | -94828 | -95320 | 492 | 2e-08 (***) | 8e-23 (***) |
| White | QUES-CAD | ACC/AHA PCE | 0.682 (0.676-0.687) | 0.672 (0.667-0.678) | 0.009 | -88506 | -88813 | 307 | 4e-05 (**) | 4e-11 (***) |
| White | QUES-CAD | FRS with lab | 0.682 (0.677-0.688) | 0.675 (0.669-0.68) | 0.008 | -94817 | -95163 | 347 | 0.002 | 1e-11 (***) |
| White | QUES-CAD | FRS without lab | 0.682 (0.677-0.688) | 0.666 (0.66-0.672) | 0.017 | -94817 | -95262 | 445 | 5e-15 (***) | 8e-24 (***) |
| White | QUES-CAD | WHO with lab | 0.683 (0.678-0.688) | 0.649 (0.644-0.655) | 0.034 | -103635 | -104495 | 860 | 2e-308 (***) | 4e-53 (***) |
| White | QUES-CAD | WHO without lab | 0.684 (0.679-0.689) | 0.656 (0.65-0.661) | 0.028 | -108679 | -109470 | 791 | 2e-308 (***) | 2e-56 (***) |
| White | QUES-CAD | Age as standalone marker | 0.684 (0.679-0.689) | 0.628 (0.622-0.633) | 0.056 | -108692 | -109721 | 1029 | 2e-308 (***) | 3e-93 (***) |
| South Asian | QUES-CAD | SCORE2 | 0.669 (0.635-0.704) | 0.668 (0.634-0.701) | 0.002 | -1654 | -1662 | 8 | 0.9 | 0.3 |
| South Asian | QUES-CAD | ACC/AHA PCE | 0.681 (0.646-0.716) | 0.678 (0.642-0.714) | 0.003 | -1485 | -1489 | 3 | 0.8 | 0.6 |
| South Asian | QUES-CAD | FRS with lab | 0.669 (0.635-0.704) | 0.676 (0.642-0.71) | -0.007 | -1654 | -1656 | 1 | 0.6 | 0.9 |
| South Asian | QUES-CAD | FRS without lab | 0.669 (0.635-0.704) | 0.665 (0.63-0.699) | 0.005 | -1654 | -1661 | 7 | 0.7 | 0.3 |
| South Asian | QUES-CAD | WHO with lab | 0.674 (0.642-0.707) | 0.656 (0.623-0.689) | 0.018 | -1823 | -1838 | 15 | 0.2 | 0.08 |
| South Asian | QUES-CAD | WHO without lab | 0.681 (0.649-0.713) | 0.649 (0.618-0.681) | 0.032 | -1904 | -1931 | 26 | 0.002 | 7e-04 (*) |
| South Asian | QUES-CAD | Age as standalone marker | 0.681 (0.649-0.713) | 0.644 (0.612-0.676) | 0.037 | -1904 | -1927 | 22 | 8e-04 (*) | 0.003 |
| Caribbean | QUES-CAD | SCORE2 | 0.59 (0.485-0.694) | 0.577 (0.474-0.68) | 0.013 | -240 | -241 | 0 | 0.7 | 0.9 |
| Caribbean | QUES-CAD | ACC/AHA PCE | 0.592 (0.483-0.7) | 0.602 (0.497-0.708) | -0.011 | -218 | -218 | -1 | 0.8 | 0.8 |
| Caribbean | QUES-CAD | FRS with lab | 0.59 (0.485-0.694) | 0.588 (0.493-0.684) | 0.001 | -240 | -240 | 0 | 1 | 0.9 |
| Caribbean | QUES-CAD | FRS without lab | 0.59 (0.485-0.694) | 0.581 (0.486-0.676) | 0.009 | -240 | -241 | 1 | 0.8 | 0.6 |
| Caribbean | QUES-CAD | WHO with lab | 0.606 (0.504-0.707) | 0.586 (0.489-0.683) | 0.019 | -261 | -263 | 2 | 0.7 | 0.6 |
| Caribbean | QUES-CAD | WHO without lab | 0.61 (0.514-0.707) | 0.574 (0.484-0.664) | 0.036 | -284 | -288 | 4 | 0.2 | 0.2 |
| Caribbean | QUES-CAD | Age as standalone marker | 0.61 (0.514-0.707) | 0.581 (0.487-0.676) | 0.029 | -284 | -287 | 3 | 0.3 | 0.2 |
| East Asian | QUES-CAD | SCORE2 | 0.714 (0.639-0.79) | 0.68 (0.605-0.755) | 0.035 | -286 | -292 | 5 | 0.2 | 0.2 |
| East Asian | QUES-CAD | ACC/AHA PCE | 0.688 (0.609-0.767) | 0.689 (0.61-0.768) | -0.001 | -255 | -256 | 1 | 1 | 0.9 |
| East Asian | QUES-CAD | FRS with lab | 0.714 (0.639-0.79) | 0.67 (0.593-0.747) | 0.045 | -286 | -292 | 6 | 0.2 | 0.1 |
| East Asian | QUES-CAD | FRS without lab | 0.714 (0.639-0.79) | 0.68 (0.602-0.758) | 0.035 | -286 | -291 | 5 | 0.2 | 0.2 |
| East Asian | QUES-CAD | WHO with lab | 0.68 (0.607-0.752) | 0.657 (0.582-0.733) | 0.022 | -335 | -339 | 4 | 0.5 | 0.4 |
| East Asian | QUES-CAD | WHO without lab | 0.66 (0.588-0.732) | 0.642 (0.574-0.711) | 0.018 | -367 | -372 | 5 | 0.4 | 0.09 |
| East Asian | QUES-CAD | Age as standalone marker | 0.66 (0.588-0.732) | 0.61 (0.532-0.687) | 0.05 | -367 | -373 | 6 | 0.05 | 0.08 |
| Black | QUES-CAD | SCORE2 | 0.731 (0.636-0.827) | 0.73 (0.652-0.807) | 0.002 | -186 | -194 | 8 | 1 | 0.06 |
| Black | QUES-CAD | ACC/AHA PCE | 0.728 (0.631-0.824) | 0.733 (0.643-0.823) | -0.006 | -179 | -185 | 6 | 0.9 | 0.1 |
| Black | QUES-CAD | FRS with lab | 0.731 (0.636-0.827) | 0.734 (0.649-0.818) | -0.002 | -186 | -193 | 6 | 0.9 | 0.1 |
| Black | QUES-CAD | FRS without lab | 0.731 (0.636-0.827) | 0.74 (0.652-0.829) | -0.009 | -186 | -190 | 4 | 0.7 | 0.3 |
| Black | QUES-CAD | WHO with lab | 0.741 (0.651-0.831) | 0.749 (0.67-0.827) | -0.007 | -202 | -210 | 9 | 0.8 | 0.06 |
| Black | QUES-CAD | WHO without lab | 0.74 (0.65-0.831) | 0.745 (0.668-0.823) | -0.005 | -204 | -209 | 5 | 0.9 | 0.1 |
| Black | QUES-CAD | Age as standalone marker | 0.74 (0.65-0.83) | 0.712 (0.618-0.806) | 0.028 | -204 | -209 | 6 | 0.4 | 0.1 |
| Other | QUES-CAD | SCORE2 | 0.676 (0.623-0.73) | 0.691 (0.639-0.742) | -0.014 | -696 | -693 | -3 | 0.5 | 0.6 |
| Other | QUES-CAD | ACC/AHA PCE | 0.686 (0.63-0.741) | 0.694 (0.642-0.746) | -0.008 | -645 | -646 | 0 | 0.6 | 0.9 |
| Other | QUES-CAD | FRS with lab | 0.676 (0.623-0.73) | 0.698 (0.647-0.749) | -0.022 | -696 | -692 | -4 | 0.3 | 0.5 |
| Other | QUES-CAD | FRS without lab | 0.676 (0.623-0.73) | 0.671 (0.618-0.724) | 0.005 | -696 | -699 | 3 | 0.8 | 0.5 |
| Other | QUES-CAD | WHO with lab | 0.687 (0.638-0.736) | 0.678 (0.628-0.727) | 0.009 | -802 | -805 | 3 | 0.7 | 0.6 |
| Other | QUES-CAD | WHO without lab | 0.692 (0.646-0.738) | 0.682 (0.636-0.728) | 0.01 | -868 | -872 | 4 | 0.6 | 0.5 |
| Other | QUES-CAD | Age as standalone marker | 0.692 (0.646-0.738) | 0.641 (0.596-0.686) | 0.051 | -868 | -879 | 11 | 0.007 | 0.04 |
| Lifelines | QUES-CAD | SCORE2 | 0.717 (0.689-0.744) | 0.743 (0.718-0.768) | -0.026 | -3132 | -3152 | 20 | 0.004 | 0.05 |
| Lifelines | QUES-CAD | ACC/AHA PCE | 0.716 (0.688-0.744) | 0.744 (0.718-0.77) | -0.028 | -3017 | -3016 | -1 | 0.002 | 0.9 |
| Lifelines | QUES-CAD | FRS with lab | 0.71 (0.681-0.738) | 0.742 (0.716-0.768) | -0.032 | -2948 | -2943 | -5 | 0.002 | 0.7 |
| Lifelines | QUES-CAD | FRS without lab | 0.71 (0.681-0.738) | 0.729 (0.701-0.756) | -0.019 | -2948 | -2954 | 6 | 0.03 | 0.6 |
| Lifelines | QUES-CAD | WHO with lab | 0.717 (0.689-0.744) | 0.731 (0.704-0.757) | -0.014 | -3132 | -3157 | 25 | 0.1 | 0.02 |
| Lifelines | QUES-CAD | WHO without lab | 0.717 (0.689-0.744) | 0.724 (0.698-0.75) | -0.007 | -3141 | -3169 | 28 | 0.4 | 0.003 |
| Lifelines | QUES-CAD | Age as standalone marker | 0.717 (0.689-0.744) | 0.686 (0.657-0.716) | 0.031 | -3141 | -3166 | 25 | 4e-04 (*) | 0.002 |

For every comparison, only participants with predictions for both models were included. Abbreviations: PL, partial log-likelihood; PLR, partial log-likelihood ratio; SCORE2, Systematic Coronary Risk Estimation 2; WHO, World Health Organization; FRS, Framingham Coronary Heart Disease Risk Score; QUES-CAD, Questionnaire-Based Evaluation for Estimating Coronary Artery Disease; ACC/AHA, American College of Cardiology/American Heart Association; CI, confidence interval.

## Supplementary Table S9B. Comparison between the C-indices, PL, and PLR of QUES-CAD and established clinical prediction algorithms and age as a standalone variable across six ethnicities and an external validation cohort using CoxGBT in women.

| **Population** | **QUES-CAD** | **Comparator model** | **C-index QUES-CAD (95% CI)** | **C-index comparator model (95% CI)** | **C-index QUES-CAD-C-index comparator model** | **PL**  **QUES-CAD** | **PL**  **comparator model** | **PLR** | **C-index**  **Bonferroni-adjusted p-value** | **PLR**  **Bonferroni-adjusted p-value** |
| --- | --- | --- | --- | --- | --- | --- | --- | --- | --- | --- |
| White | QUES-CAD | SCORE2 | 0.735 (0.728-0.742) | 0.723 (0.716-0.73) | 0.012 | -51774 | -52215 | 441 | 1e-05 (***) | 8e-21 (***) |
| White | QUES-CAD | ACC/AHA PCE | 0.736 (0.729-0.744) | 0.728 (0.721-0.735) | 0.008 | -47143 | -47535 | 392 | 0.002 | 3e-19 (***) |
| White | QUES-CAD | FRS with lab | 0.735 (0.728-0.742) | 0.708 (0.7-0.715) | 0.027 | -51638 | -52199 | 560 | 6e-13 (***) | 3e-25 (***) |
| White | QUES-CAD | FRS without lab | 0.735 (0.728-0.742) | 0.712 (0.705-0.719) | 0.023 | -51638 | -52186 | 547 | 3e-11 (***) | 4e-27 (***) |
| White | QUES-CAD | WHO with lab | 0.737 (0.73-0.744) | 0.71 (0.703-0.717) | 0.027 | -57233 | -58108 | 876 | 2e-308 (***) | 5e-64 (***) |
| White | QUES-CAD | WHO without lab | 0.737 (0.73-0.744) | 0.713 (0.706-0.72) | 0.024 | -60116 | -60908 | 792 | 2e-308 (***) | 1e-56 (***) |
| White | QUES-CAD | Age as standalone marker | 0.737 (0.731-0.744) | 0.676 (0.669-0.684) | 0.061 | -60296 | -61168 | 872 | 2e-308 (***) | 8e-69 (***) |
| South Asian | QUES-CAD | SCORE2 | 0.741 (0.695-0.786) | 0.744 (0.694-0.795) | -0.004 | -688 | -687 | -1 | 0.9 | 0.9 |
| South Asian | QUES-CAD | ACC/AHA PCE | 0.745 (0.697-0.792) | 0.757 (0.707-0.806) | -0.012 | -630 | -627 | -2 | 0.5 | 0.8 |
| South Asian | QUES-CAD | FRS with lab | 0.738 (0.691-0.785) | 0.754 (0.707-0.802) | -0.017 | -665 | -664 | -1 | 0.4 | 0.9 |
| South Asian | QUES-CAD | FRS without lab | 0.738 (0.691-0.785) | 0.75 (0.702-0.799) | -0.013 | -665 | -663 | -2 | 0.5 | 0.8 |
| South Asian | QUES-CAD | WHO with lab | 0.725 (0.679-0.77) | 0.713 (0.662-0.765) | 0.011 | -771 | -775 | 3 | 0.6 | 0.6 |
| South Asian | QUES-CAD | WHO without lab | 0.725 (0.678-0.771) | 0.706 (0.658-0.754) | 0.019 | -799 | -812 | 13 | 0.2 | 0.03 |
| South Asian | QUES-CAD | Age as standalone marker | 0.728 (0.682-0.773) | 0.679 (0.63-0.728) | 0.049 | -822 | -833 | 11 | 4e-04 (*) | 0.04 |
| Caribbean | QUES-CAD | SCORE2 | 0.746 (0.686-0.805) | 0.75 (0.692-0.808) | -0.004 | -392 | -395 | 4 | 0.9 | 0.5 |
| Caribbean | QUES-CAD | ACC/AHA PCE | 0.74 (0.678-0.802) | 0.742 (0.677-0.808) | -0.002 | -369 | -374 | 5 | 0.9 | 0.3 |
| Caribbean | QUES-CAD | FRS with lab | 0.748 (0.687-0.808) | 0.777 (0.722-0.833) | -0.03 | -383 | -385 | 2 | 0.4 | 0.8 |
| Caribbean | QUES-CAD | FRS without lab | 0.748 (0.687-0.808) | 0.777 (0.722-0.831) | -0.029 | -383 | -386 | 3 | 0.3 | 0.7 |
| Caribbean | QUES-CAD | WHO with lab | 0.761 (0.706-0.816) | 0.763 (0.708-0.817) | -0.002 | -439 | -446 | 7 | 0.9 | 0.3 |
| Caribbean | QUES-CAD | WHO without lab | 0.764 (0.709-0.82) | 0.733 (0.675-0.791) | 0.032 | -433 | -446 | 12 | 0.2 | 0.05 |
| Caribbean | QUES-CAD | Age as standalone marker | 0.762 (0.708-0.817) | 0.671 (0.601-0.741) | 0.091 | -442 | -458 | 16 | 0.003 | 0.02 |
| East Asian | QUES-CAD | SCORE2 | 0.801 (0.723-0.88) | 0.822 (0.763-0.881) | -0.021 | -215 | -224 | 8 | 0.4 | 0.1 |
| East Asian | QUES-CAD | ACC/AHA PCE | 0.809 (0.725-0.892) | 0.823 (0.754-0.891) | -0.014 | -193 | -195 | 2 | 0.6 | 0.7 |
| East Asian | QUES-CAD | FRS with lab | 0.801 (0.723-0.88) | 0.824 (0.766-0.882) | -0.022 | -215 | -222 | 7 | 0.5 | 0.2 |
| East Asian | QUES-CAD | FRS without lab | 0.801 (0.723-0.88) | 0.823 (0.769-0.877) | -0.022 | -215 | -223 | 8 | 0.5 | 0.2 |
| East Asian | QUES-CAD | WHO with lab | 0.803 (0.729-0.878) | 0.814 (0.75-0.878) | -0.01 | -238 | -250 | 12 | 0.7 | 0.03 |
| East Asian | QUES-CAD | WHO without lab | 0.798 (0.725-0.871) | 0.781 (0.719-0.842) | 0.017 | -248 | -262 | 15 | 0.4 | 0.009 |
| East Asian | QUES-CAD | Age as standalone marker | 0.798 (0.724-0.871) | 0.78 (0.715-0.845) | 0.017 | -248 | -252 | 4 | 0.3 | 0.4 |
| Black | QUES-CAD | SCORE2 | 0.672 (0.521-0.824) | 0.742 (0.598-0.885) | -0.069 | -117 | -111 | -5 | 0.02 | 0.08 |
| Black | QUES-CAD | ACC/AHA PCE | 0.706 (0.552-0.86) | 0.746 (0.592-0.9) | -0.04 | -94 | -86 | -7 | 0.5 | 0.2 |
| Black | QUES-CAD | FRS with lab | 0.672 (0.521-0.824) | 0.753 (0.616-0.889) | -0.081 | -116 | -111 | -5 | 0.02 | 0.09 |
| Black | QUES-CAD | FRS without lab | 0.672 (0.521-0.824) | 0.753 (0.62-0.886) | -0.081 | -116 | -110 | -7 | 0.03 | 0.1 |
| Black | QUES-CAD | WHO with lab | 0.673 (0.547-0.798) | 0.666 (0.527-0.804) | 0.007 | -147 | -147 | -1 | 0.9 | 0.6 |
| Black | QUES-CAD | WHO without lab | 0.658 (0.529-0.787) | 0.665 (0.544-0.787) | -0.007 | -163 | -164 | 1 | 0.8 | 0.5 |
| Black | QUES-CAD | Age as standalone marker | 0.658 (0.529-0.788) | 0.616 (0.481-0.751) | 0.043 | -163 | -164 | 1 | 0.2 | 0.4 |
| Other | QUES-CAD | SCORE2 | 0.747 (0.68-0.813) | 0.781 (0.725-0.837) | -0.035 | -441 | -438 | -2 | 0.1 | 0.6 |
| Other | QUES-CAD | ACC/AHA PCE | 0.765 (0.7-0.831) | 0.794 (0.737-0.851) | -0.029 | -396 | -393 | -3 | 0.2 | 0.5 |
| Other | QUES-CAD | FRS with lab | 0.746 (0.68-0.813) | 0.78 (0.719-0.841) | -0.034 | -440 | -432 | -8 | 0.2 | 0.2 |
| Other | QUES-CAD | FRS without lab | 0.746 (0.68-0.813) | 0.781 (0.722-0.84) | -0.035 | -440 | -436 | -4 | 0.2 | 0.5 |
| Other | QUES-CAD | WHO with lab | 0.747 (0.685-0.81) | 0.766 (0.704-0.829) | -0.019 | -478 | -478 | 0 | 0.4 | 0.9 |
| Other | QUES-CAD | WHO without lab | 0.748 (0.686-0.811) | 0.76 (0.704-0.817) | -0.012 | -480 | -486 | 5 | 0.6 | 0.3 |
| Other | QUES-CAD | Age as standalone marker | 0.752 (0.69-0.814) | 0.706 (0.64-0.773) | 0.046 | -487 | -494 | 7 | 0.03 | 0.2 |
| Lifelines | QUES-CAD | SCORE2 | 0.774 (0.744-0.804) | 0.779 (0.75-0.809) | -0.006 | -2397 | -2446 | 49 | 0.6 | 3e-05 (**) |
| Lifelines | QUES-CAD | ACC/AHA PCE | 0.771 (0.739-0.804) | 0.784 (0.753-0.814) | -0.012 | -2151 | -2196 | 45 | 0.3 | 8e-05 (**) |
| Lifelines | QUES-CAD | FRS with lab | 0.768 (0.737-0.8) | 0.778 (0.747-0.808) | -0.009 | -2278 | -2302 | 24 | 0.5 | 0.04 |
| Lifelines | QUES-CAD | FRS without lab | 0.768 (0.737-0.8) | 0.766 (0.735-0.796) | 0.003 | -2278 | -2319 | 41 | 0.8 | 4e-04 (*) |
| Lifelines | QUES-CAD | WHO with lab | 0.774 (0.744-0.804) | 0.764 (0.732-0.795) | 0.01 | -2397 | -2455 | 58 | 0.4 | 6e-06 (***) |
| Lifelines | QUES-CAD | WHO without lab | 0.774 (0.744-0.804) | 0.759 (0.728-0.79) | 0.015 | -2398 | -2458 | 61 | 0.2 | 1e-06 (***) |
| Lifelines | QUES-CAD | Age as standalone marker | 0.773 (0.743-0.803) | 0.727 (0.693-0.76) | 0.046 | -2408 | -2447 | 38 | 7e-05 (**) | 2e-04 (**) |

For every comparison, only participants with predictions for both models were included. Abbreviations: PL, partial log-likelihood; PLR, partial log-likelihood ratio; SCORE2, Systematic Coronary Risk Estimation 2; WHO, World Health Organization; FRS, Framingham Coronary Heart Disease Risk Score; QUES-CAD, Questionnaire-Based Evaluation for Estimating Coronary Artery Disease; ACC/AHA, American College of Cardiology/American Heart Association; CI, confidence interval.

## Supplementary Table S9C. Comparison between the C-indices, PL, and PLR of QUES-CAD and established clinical prediction algorithms and age as a standalone variable across six ethnicities and an external validation cohort using CoxPH in male subjects.

| **Population** | **QUES-CAD** | **Comparator model** | **C-index QUES-CAD (95% CI)** | **C-index comparator model (95% CI)** | **C-index QUES-CAD-C-index comparator model** | **PL**  **QUES-CAD** | **PL**  **comparator model** | **PLR** | **C-index**  **Bonferroni-adjusted p-value** | **PLR**  **Bonferroni-adjusted p-value** |
| --- | --- | --- | --- | --- | --- | --- | --- | --- | --- | --- |
| White | QUES-CAD | SCORE2 | 0.687 (0.681-0.693) | 0.669 (0.663-0.674) | 0.018 | -95250 | -95320 | 70 | 3e-13 (***) | 0.1 |
| White | QUES-CAD | ACC/AHA PCE | 0.686 (0.681-0.692) | 0.672 (0.667-0.678) | 0.014 | -88901 | -88813 | -88 | 3e-09 (***) | 0.05 |
| White | QUES-CAD | FRS with lab | 0.687 (0.681-0.693) | 0.675 (0.669-0.68) | 0.012 | -95239 | -95163 | -75 | 2e-06 (***) | 0.1 |
| White | QUES-CAD | FRS without lab | 0.687 (0.681-0.693) | 0.666 (0.66-0.672) | 0.021 | -95239 | -95262 | 24 | 2e-308 (***) | 0.6 |
| White | QUES-CAD | WHO with lab | 0.688 (0.682-0.693) | 0.649 (0.644-0.655) | 0.038 | -104093 | -104495 | 402 | 2e-308 (***) | 4e-15 (***) |
| White | QUES-CAD | WHO without lab | 0.688 (0.683-0.694) | 0.656 (0.65-0.661) | 0.033 | -109158 | -109470 | 312 | 2e-308 (***) | 7e-12 (***) |
| White | QUES-CAD | Age as standalone marker | 0.688 (0.683-0.694) | 0.628 (0.622-0.633) | 0.061 | -109171 | -109721 | 550 | 2e-308 (***) | 1e-30 (***) |
| South Asian | QUES-CAD | SCORE2 | 0.679 (0.645-0.713) | 0.668 (0.634-0.701) | 0.011 | -1661 | -1662 | 1 | 0.4 | 0.9 |
| South Asian | QUES-CAD | ACC/AHA PCE | 0.688 (0.652-0.723) | 0.678 (0.642-0.714) | 0.01 | -1494 | -1489 | -5 | 0.5 | 0.5 |
| South Asian | QUES-CAD | FRS with lab | 0.679 (0.645-0.713) | 0.676 (0.642-0.71) | 0.003 | -1661 | -1656 | -6 | 0.8 | 0.4 |
| South Asian | QUES-CAD | FRS without lab | 0.679 (0.645-0.713) | 0.665 (0.63-0.699) | 0.014 | -1661 | -1661 | 0 | 0.3 | 1 |
| South Asian | QUES-CAD | WHO with lab | 0.685 (0.653-0.717) | 0.656 (0.623-0.689) | 0.029 | -1831 | -1838 | 7 | 0.05 | 0.3 |
| South Asian | QUES-CAD | WHO without lab | 0.691 (0.659-0.723) | 0.649 (0.618-0.681) | 0.041 | -1914 | -1931 | 17 | 8e-04 (*) | 0.009 |
| South Asian | QUES-CAD | Age as standalone marker | 0.691 (0.659-0.723) | 0.644 (0.612-0.676) | 0.047 | -1914 | -1927 | 13 | 6e-04 (*) | 0.07 |
| Caribbean | QUES-CAD | SCORE2 | 0.584 (0.484-0.684) | 0.577 (0.474-0.68) | 0.007 | -241 | -241 | -1 | 0.9 | 0.6 |
| Caribbean | QUES-CAD | ACC/AHA PCE | 0.584 (0.479-0.688) | 0.602 (0.497-0.708) | -0.019 | -219 | -218 | -1 | 0.6 | 0.3 |
| Caribbean | QUES-CAD | FRS with lab | 0.584 (0.484-0.684) | 0.588 (0.493-0.684) | -0.005 | -241 | -240 | -1 | 0.9 | 0.5 |
| Caribbean | QUES-CAD | FRS without lab | 0.584 (0.484-0.684) | 0.581 (0.486-0.676) | 0.003 | -241 | -241 | 0 | 0.9 | 0.9 |
| Caribbean | QUES-CAD | WHO with lab | 0.597 (0.5-0.694) | 0.586 (0.489-0.683) | 0.011 | -263 | -263 | 0 | 0.8 | 0.9 |
| Caribbean | QUES-CAD | WHO without lab | 0.604 (0.512-0.696) | 0.574 (0.484-0.664) | 0.03 | -286 | -288 | 2 | 0.4 | 0.3 |
| Caribbean | QUES-CAD | Age as standalone marker | 0.604 (0.512-0.696) | 0.581 (0.487-0.676) | 0.023 | -286 | -287 | 1 | 0.5 | 0.5 |
| East Asian | QUES-CAD | SCORE2 | 0.697 (0.615-0.779) | 0.68 (0.605-0.755) | 0.017 | -290 | -292 | 1 | 0.6 | 0.7 |
| East Asian | QUES-CAD | ACC/AHA PCE | 0.672 (0.585-0.76) | 0.689 (0.61-0.768) | -0.017 | -258 | -256 | -2 | 0.6 | 0.5 |
| East Asian | QUES-CAD | FRS with lab | 0.697 (0.615-0.779) | 0.67 (0.593-0.747) | 0.027 | -290 | -292 | 2 | 0.5 | 0.5 |
| East Asian | QUES-CAD | FRS without lab | 0.697 (0.615-0.779) | 0.68 (0.602-0.758) | 0.017 | -290 | -291 | 1 | 0.6 | 0.8 |
| East Asian | QUES-CAD | WHO with lab | 0.667 (0.59-0.743) | 0.657 (0.582-0.733) | 0.009 | -338 | -339 | 1 | 0.8 | 0.9 |
| East Asian | QUES-CAD | WHO without lab | 0.655 (0.581-0.729) | 0.642 (0.574-0.711) | 0.013 | -369 | -372 | 3 | 0.6 | 0.2 |
| East Asian | QUES-CAD | Age as standalone marker | 0.655 (0.581-0.729) | 0.61 (0.532-0.687) | 0.046 | -369 | -373 | 4 | 0.2 | 0.2 |
| Black | QUES-CAD | SCORE2 | 0.756 (0.654-0.857) | 0.73 (0.652-0.807) | 0.026 | -186 | -194 | 8 | 0.6 | 0.05 |
| Black | QUES-CAD | ACC/AHA PCE | 0.754 (0.65-0.857) | 0.733 (0.643-0.823) | 0.021 | -178 | -185 | 6 | 0.7 | 0.1 |
| Black | QUES-CAD | FRS with lab | 0.756 (0.654-0.857) | 0.734 (0.649-0.818) | 0.022 | -186 | -193 | 6 | 0.6 | 0.1 |
| Black | QUES-CAD | FRS without lab | 0.756 (0.654-0.857) | 0.74 (0.652-0.829) | 0.015 | -186 | -190 | 4 | 0.7 | 0.2 |
| Black | QUES-CAD | WHO with lab | 0.765 (0.669-0.86) | 0.749 (0.67-0.827) | 0.016 | -202 | -210 | 8 | 0.7 | 0.06 |
| Black | QUES-CAD | WHO without lab | 0.765 (0.67-0.861) | 0.745 (0.668-0.823) | 0.02 | -204 | -209 | 5 | 0.6 | 0.1 |
| Black | QUES-CAD | Age as standalone marker | 0.765 (0.669-0.86) | 0.712 (0.618-0.806) | 0.053 | -204 | -209 | 5 | 0.3 | 0.2 |
| Other | QUES-CAD | SCORE2 | 0.677 (0.62-0.733) | 0.691 (0.639-0.742) | -0.014 | -697 | -693 | -4 | 0.6 | 0.5 |
| Other | QUES-CAD | ACC/AHA PCE | 0.687 (0.629-0.744) | 0.694 (0.642-0.746) | -0.008 | -647 | -646 | -1 | 0.7 | 0.8 |
| Other | QUES-CAD | FRS with lab | 0.677 (0.62-0.733) | 0.698 (0.647-0.749) | -0.022 | -697 | -692 | -5 | 0.4 | 0.4 |
| Other | QUES-CAD | FRS without lab | 0.677 (0.62-0.733) | 0.671 (0.618-0.724) | 0.005 | -697 | -699 | 2 | 0.8 | 0.7 |
| Other | QUES-CAD | WHO with lab | 0.682 (0.63-0.734) | 0.678 (0.628-0.727) | 0.005 | -805 | -805 | 0 | 0.9 | 1 |
| Other | QUES-CAD | WHO without lab | 0.689 (0.64-0.738) | 0.682 (0.636-0.728) | 0.007 | -871 | -872 | 1 | 0.7 | 0.9 |
| Other | QUES-CAD | Age as standalone marker | 0.689 (0.64-0.738) | 0.641 (0.596-0.686) | 0.048 | -871 | -879 | 7 | 0.03 | 0.2 |
| Lifelines | QUES-CAD | SCORE2 | 0.718 (0.691-0.745) | 0.743 (0.718-0.768) | -0.025 | -3166 | -3152 | -14 | 0.009 | 0.07 |
| Lifelines | QUES-CAD | ACC/AHA PCE | 0.718 (0.691-0.746) | 0.744 (0.718-0.77) | -0.026 | -3039 | -3016 | -22 | 0.007 | 0.03 |
| Lifelines | QUES-CAD | FRS with lab | 0.711 (0.683-0.739) | 0.742 (0.716-0.768) | -0.031 | -2966 | -2943 | -23 | 0.004 | 0.04 |
| Lifelines | QUES-CAD | FRS without lab | 0.711 (0.683-0.739) | 0.729 (0.701-0.756) | -0.018 | -2966 | -2954 | -12 | 0.05 | 0.2 |
| Lifelines | QUES-CAD | WHO with lab | 0.718 (0.691-0.745) | 0.731 (0.704-0.757) | -0.013 | -3166 | -3157 | -9 | 0.2 | 0.3 |
| Lifelines | QUES-CAD | WHO without lab | 0.718 (0.691-0.745) | 0.724 (0.698-0.75) | -0.006 | -3176 | -3169 | -7 | 0.5 | 0.4 |
| Lifelines | QUES-CAD | Age as standalone marker | 0.718 (0.691-0.745) | 0.686 (0.657-0.716) | 0.032 | -3176 | -3166 | -9 | 0.003 | 0.3 |

For every comparison, only participants with predictions for both models were included. Abbreviations: PL, partial log-likelihood; PLR, partial log-likelihood ratio; SCORE2, Systematic Coronary Risk Estimation 2; WHO, World Health Organization; FRS, Framingham Coronary Heart Disease Risk Score; QUES-CAD, Questionnaire-Based Evaluation for Estimating Coronary Artery Disease; ACC/AHA, American College of Cardiology/American Heart Association; CI, confidence interval.

## Supplementary Table S9D. Comparison between the C-indices, PL, and PLR of QUES-CAD and established clinical prediction algorithms and age as a standalone variable across six ethnicities and an external validation cohort using CoxPH in women.

| **Population** | **QUES-CAD** | **Comparator model** | **C-index QUES-CAD (95% CI)** | **C-index comparator model (95% CI)** | **C-index QUES-CAD-C-index comparator model** | **PL**  **QUES-CAD** | **PL**  **comparator model** | **PLR** | **C-index**  **Bonferroni-adjusted p-value** | **PLR**  **Bonferroni-adjusted p-value** |
| --- | --- | --- | --- | --- | --- | --- | --- | --- | --- | --- |
| White | QUES-CAD | SCORE2 | 0.74 (0.733-0.747) | 0.723 (0.716-0.73) | 0.017 | -52459 | -52215 | -244 | 2e-09 (***) | 4e-09 (***) |
| White | QUES-CAD | ACC/AHA PCE | 0.741 (0.734-0.748) | 0.728 (0.721-0.735) | 0.013 | -47785 | -47535 | -250 | 3e-06 (***) | 2e-10 (***) |
| White | QUES-CAD | FRS with lab | 0.74 (0.733-0.747) | 0.708 (0.7-0.715) | 0.033 | -52322 | -52199 | -123 | 2e-308 (***) | 0.006 |
| White | QUES-CAD | FRS without lab | 0.74 (0.733-0.747) | 0.712 (0.705-0.719) | 0.028 | -52322 | -52186 | -136 | 2e-16 (***) | 0.001 |
| White | QUES-CAD | WHO with lab | 0.742 (0.735-0.748) | 0.71 (0.703-0.717) | 0.032 | -57999 | -58108 | 110 | 2e-308 (***) | 0.005 |
| White | QUES-CAD | WHO without lab | 0.742 (0.735-0.749) | 0.713 (0.706-0.72) | 0.029 | -60920 | -60908 | -12 | 2e-308 (***) | 0.8 |
| White | QUES-CAD | Age as standalone marker | 0.742 (0.735-0.749) | 0.676 (0.669-0.684) | 0.066 | -61111 | -61168 | 57 | 2e-308 (***) | 0.2 |
| South Asian | QUES-CAD | SCORE2 | 0.749 (0.704-0.794) | 0.744 (0.694-0.795) | 0.004 | -699 | -687 | -12 | 0.8 | 0.2 |
| South Asian | QUES-CAD | ACC/AHA PCE | 0.746 (0.699-0.794) | 0.757 (0.707-0.806) | -0.01 | -641 | -627 | -13 | 0.6 | 0.1 |
| South Asian | QUES-CAD | FRS with lab | 0.745 (0.699-0.791) | 0.754 (0.707-0.802) | -0.01 | -676 | -664 | -11 | 0.6 | 0.2 |
| South Asian | QUES-CAD | FRS without lab | 0.745 (0.699-0.791) | 0.75 (0.702-0.799) | -0.005 | -676 | -663 | -12 | 0.8 | 0.1 |
| South Asian | QUES-CAD | WHO with lab | 0.733 (0.688-0.777) | 0.713 (0.662-0.765) | 0.019 | -781 | -775 | -7 | 0.3 | 0.4 |
| South Asian | QUES-CAD | WHO without lab | 0.733 (0.688-0.778) | 0.706 (0.658-0.754) | 0.027 | -809 | -812 | 3 | 0.08 | 0.6 |
| South Asian | QUES-CAD | Age as standalone marker | 0.736 (0.692-0.781) | 0.679 (0.63-0.728) | 0.057 | -833 | -833 | 1 | 2e-04 (**) | 0.9 |
| Caribbean | QUES-CAD | SCORE2 | 0.744 (0.683-0.805) | 0.75 (0.692-0.808) | -0.006 | -394 | -395 | 1 | 0.8 | 0.8 |
| Caribbean | QUES-CAD | ACC/AHA PCE | 0.74 (0.677-0.803) | 0.742 (0.677-0.808) | -0.003 | -372 | -374 | 2 | 0.9 | 0.6 |
| Caribbean | QUES-CAD | FRS with lab | 0.747 (0.685-0.809) | 0.777 (0.722-0.833) | -0.03 | -385 | -385 | -1 | 0.4 | 0.9 |
| Caribbean | QUES-CAD | FRS without lab | 0.747 (0.685-0.809) | 0.777 (0.722-0.831) | -0.029 | -385 | -386 | 0 | 0.3 | 1 |
| Caribbean | QUES-CAD | WHO with lab | 0.758 (0.702-0.814) | 0.763 (0.708-0.817) | -0.005 | -443 | -446 | 3 | 0.9 | 0.7 |
| Caribbean | QUES-CAD | WHO without lab | 0.763 (0.707-0.82) | 0.733 (0.675-0.791) | 0.031 | -438 | -446 | 8 | 0.3 | 0.2 |
| Caribbean | QUES-CAD | Age as standalone marker | 0.76 (0.705-0.816) | 0.671 (0.601-0.741) | 0.089 | -447 | -458 | 11 | 0.005 | 0.1 |
| East Asian | QUES-CAD | SCORE2 | 0.809 (0.737-0.881) | 0.822 (0.763-0.881) | -0.013 | -225 | -224 | -1 | 0.6 | 0.7 |
| East Asian | QUES-CAD | ACC/AHA PCE | 0.817 (0.741-0.893) | 0.823 (0.754-0.891) | -0.006 | -201 | -195 | -7 | 0.8 | 0.2 |
| East Asian | QUES-CAD | FRS with lab | 0.809 (0.737-0.881) | 0.824 (0.766-0.882) | -0.015 | -225 | -222 | -3 | 0.7 | 0.4 |
| East Asian | QUES-CAD | FRS without lab | 0.809 (0.737-0.881) | 0.823 (0.769-0.877) | -0.014 | -225 | -223 | -2 | 0.7 | 0.6 |
| East Asian | QUES-CAD | WHO with lab | 0.813 (0.745-0.881) | 0.814 (0.75-0.878) | -0.001 | -247 | -250 | 2 | 1 | 0.4 |
| East Asian | QUES-CAD | WHO without lab | 0.808 (0.74-0.875) | 0.781 (0.719-0.842) | 0.027 | -257 | -262 | 5 | 0.2 | 0.07 |
| East Asian | QUES-CAD | Age as standalone marker | 0.807 (0.739-0.875) | 0.78 (0.715-0.845) | 0.027 | -257 | -252 | -5 | 0.2 | 0.2 |
| Black | QUES-CAD | SCORE2 | 0.679 (0.53-0.828) | 0.742 (0.598-0.885) | -0.062 | -118 | -111 | -6 | 0.01 | 0.06 |
| Black | QUES-CAD | ACC/AHA PCE | 0.706 (0.553-0.859) | 0.746 (0.592-0.9) | -0.04 | -95 | -86 | -9 | 0.4 | 0.1 |
| Black | QUES-CAD | FRS with lab | 0.679 (0.53-0.828) | 0.753 (0.616-0.889) | -0.074 | -117 | -111 | -6 | 0.02 | 0.06 |
| Black | QUES-CAD | FRS without lab | 0.679 (0.53-0.828) | 0.753 (0.62-0.886) | -0.074 | -117 | -110 | -8 | 0.03 | 0.08 |
| Black | QUES-CAD | WHO with lab | 0.665 (0.538-0.791) | 0.666 (0.527-0.804) | -0.001 | -149 | -147 | -3 | 1 | 0.1 |
| Black | QUES-CAD | WHO without lab | 0.651 (0.521-0.78) | 0.665 (0.544-0.787) | -0.015 | -165 | -164 | -1 | 0.6 | 0.2 |
| Black | QUES-CAD | Age as standalone marker | 0.651 (0.522-0.781) | 0.616 (0.481-0.751) | 0.036 | -165 | -164 | -1 | 0.2 | 0.5 |
| Other | QUES-CAD | SCORE2 | 0.762 (0.701-0.823) | 0.781 (0.725-0.837) | -0.019 | -450 | -438 | -12 | 0.4 | 0.02 |
| Other | QUES-CAD | ACC/AHA PCE | 0.78 (0.72-0.841) | 0.794 (0.737-0.851) | -0.014 | -407 | -393 | -13 | 0.5 | 0.02 |
| Other | QUES-CAD | FRS with lab | 0.762 (0.701-0.824) | 0.78 (0.719-0.841) | -0.018 | -450 | -432 | -18 | 0.5 | 0.01 |
| Other | QUES-CAD | FRS without lab | 0.762 (0.701-0.824) | 0.781 (0.722-0.84) | -0.019 | -450 | -436 | -14 | 0.5 | 0.03 |
| Other | QUES-CAD | WHO with lab | 0.76 (0.702-0.819) | 0.766 (0.704-0.829) | -0.006 | -488 | -478 | -10 | 0.8 | 0.05 |
| Other | QUES-CAD | WHO without lab | 0.762 (0.703-0.82) | 0.76 (0.704-0.817) | 0.002 | -490 | -486 | -5 | 1 | 0.2 |
| Other | QUES-CAD | Age as standalone marker | 0.765 (0.707-0.823) | 0.706 (0.64-0.773) | 0.059 | -496 | -494 | -3 | 0.02 | 0.6 |
| Lifelines | QUES-CAD | SCORE2 | 0.778 (0.749-0.808) | 0.779 (0.75-0.809) | -0.001 | -2438 | -2446 | 8 | 0.9 | 0.2 |
| Lifelines | QUES-CAD | ACC/AHA PCE | 0.777 (0.745-0.809) | 0.784 (0.753-0.814) | -0.007 | -2188 | -2196 | 8 | 0.5 | 0.3 |
| Lifelines | QUES-CAD | FRS with lab | 0.772 (0.741-0.804) | 0.778 (0.747-0.808) | -0.005 | -2313 | -2302 | -11 | 0.7 | 0.3 |
| Lifelines | QUES-CAD | FRS without lab | 0.772 (0.741-0.804) | 0.766 (0.735-0.796) | 0.007 | -2313 | -2319 | 6 | 0.6 | 0.5 |
| Lifelines | QUES-CAD | WHO with lab | 0.778 (0.749-0.808) | 0.764 (0.732-0.795) | 0.015 | -2438 | -2455 | 17 | 0.1 | 0.02 |
| Lifelines | QUES-CAD | WHO without lab | 0.778 (0.749-0.808) | 0.759 (0.728-0.79) | 0.019 | -2439 | -2458 | 20 | 0.03 | 0.005 |
| Lifelines | QUES-CAD | Age as standalone marker | 0.778 (0.748-0.807) | 0.727 (0.693-0.76) | 0.051 | -2449 | -2447 | -2 | 3e-06 (***) | 0.8 |

For every comparison, only participants with predictions for both models were included. Abbreviations: PL, partial log-likelihood; PLR, partial log-likelihood ratio; SCORE2, Systematic Coronary Risk Estimation 2; WHO, World Health Organization; FRS, Framingham Coronary Heart Disease Risk Score; QUES-CAD, Questionnaire-Based Evaluation for Estimating Coronary Artery Disease; ACC/AHA, American College of Cardiology/American Heart Association; CI, confidence interval.

## Supplementary Figure S3. Predictive abilities of the several Coronary Artery Disease forecasting models constructed in this study for men (left panel) and women (right panel).

The several symbols represent each different model (i.e., questionnaire-only / QUES-CAD, Questionnaire & Measurement-based variables [or variables that require prior medical examination], and Questionnaire, measurement-based variables (or variables that require prior medical examination), and other biomarkers). Each color-symbol combination refers to a specific model and population, explained in detail in the lateral panel. The AUC and 95% CI are presented for all models as horizontal lines. Abbreviations: CoxPH; Cox proportional hazards; CoxGBT, Cox gradient boosting; QUES-CAD, Questionnaire-Based Evaluation for Estimating Coronary Artery Disease.

# Questionnaire & Measurement-based variables (or variables that require prior medical examination)

## Supplementary Figure S4A. Questionnaire & Measurement-based variables (or variables that require prior medical examination)-based feature importance with coronary artery disease prediction in women.

## Supplementary Figure S4B. Questionnaire & Measurement-based variables (or variables that require prior medical examination)-based features’ hazard ratios for coronary artery disease prediction in women.

## Supplementary Figure S4C. Questionnaire & Measurement-based variables (or variables that require prior medical examination)-based feature importance with coronary artery disease prediction in men.

## Supplementary Figure S4D. Questionnaire & Measurement-based variables (or variables that require prior medical examination)-based features’ hazard ratios for coronary artery disease prediction in men.

## Supplementary Table S10. Diagnostic metrics of the developed models containing questionnaire & measurement-based variables (or variables that require prior medical examination) features.

| **Sex** | **Model** | **Ethnicity** | **C-index** | **N** | **Low risk** | **High risk** | **Sensitivity (95% CI)** | **Specificity (95% CI)** | **PPV (95% CI)** | **NPV (95% CI)** | **Threshold** | **10-year incidence** |
| --- | --- | --- | --- | --- | --- | --- | --- | --- | --- | --- | --- | --- |
| men | CoxPH | White | 0.7 (0.695-0.705) | 177301 | 130152 | 47149 | 55 (54-56) | 75 (74-75) | 8 (8-8) | 98 (98-98) | 0.043 | 0.037 |
| men | CoxPH | South Asian | 0.724 (0.696-0.751) | 3411 | 2375 | 1036 | 57 (51-65) | 72 (70-73) | 13 (11-15) | 96 (95-97) | 0.043 | 0.066 |
| men | CoxPH | Caribbean | 0.705 (0.641-0.77) | 1538 | 1249 | 289 | 46 (33-60) | 82 (80-84) | 6 (4-9) | 98 (98-99) | 0.043 | 0.026 |
| men | CoxPH | East Asian | 0.658 (0.595-0.722) | 1572 | 1249 | 323 | 44 (29-55) | 80 (78-82) | 6 (4-8) | 98 (98-99) | 0.043 | 0.026 |
| men | CoxPH | Black | 0.768 (0.694-0.841) | 1577 | 1294 | 283 | 55 (33-72) | 83 (81-85) | 6 (3-8) | 99 (98-99) | 0.043 | 0.019 |
| men | CoxPH | Other | 0.728 (0.692-0.764) | 2779 | 2092 | 687 | 56 (49-64) | 76 (75-78) | 8 (6-9) | 98 (98-98) | 0.043 | 0.033 |
| men | CoxPH | Lifelines | 0.711 (0.693-0.728) | 39651 | 31687 | 7964 | 42 (38-45) | 81 (80-81) | 8 (7-9) | 97 (97-97) | 0.043 | 0.04 |
| men | CoxGBT | White | 0.697 (0.692-0.701) | 177301 | 130142 | 47159 | 55 (54-56) | 75 (74-75) | 8 (8-8) | 98 (98-98) | 0.269 | 0.037 |
| men | CoxGBT | South Asian | 0.711 (0.683-0.739) | 3411 | 2489 | 922 | 53 (47-60) | 75 (73-76) | 13 (11-15) | 96 (95-96) | 0.269 | 0.066 |
| men | CoxGBT | Caribbean | 0.708 (0.642-0.773) | 1538 | 1235 | 303 | 48 (31-65) | 81 (79-83) | 7 (4-9) | 98 (98-99) | 0.269 | 0.026 |
| men | CoxGBT | East Asian | 0.674 (0.617-0.732) | 1572 | 1262 | 310 | 47 (30-61) | 81 (79-83) | 6 (4-9) | 98 (98-99) | 0.269 | 0.026 |
| men | CoxGBT | Black | 0.753 (0.677-0.828) | 1577 | 1316 | 261 | 45 (26-61) | 84 (82-86) | 5 (3-8) | 99 (98-99) | 0.269 | 0.019 |
| men | CoxGBT | Other | 0.722 (0.687-0.757) | 2779 | 2130 | 649 | 55 (45-63) | 78 (76-79) | 8 (6-10) | 98 (98-98) | 0.269 | 0.033 |
| men | CoxGBT | Lifelines | 0.718 (0.7-0.735) | 39651 | 33112 | 6539 | 39 (36-42) | 84 (84-85) | 9 (9-10) | 97 (97-97) | 0.269 | 0.04 |
| women | CoxPH | White | 0.756 (0.75-0.763) | 214108 | 201214 | 12894 | 29 (27-31) | 94 (94-94) | 6 (6-7) | 99 (99-99) | 0.035 | 0.014 |
| women | CoxPH | South Asian | 0.746 (0.699-0.793) | 2977 | 2757 | 220 | 36 (23-48) | 93 (93-94) | 10 (6-14) | 99 (98-99) | 0.035 | 0.02 |
| women | CoxPH | Caribbean | 0.81 (0.764-0.857) | 2645 | 2482 | 163 | 36 (21-54) | 94 (93-95) | 8 (4-12) | 99 (99-99) | 0.035 | 0.013 |
| women | CoxPH | East Asian | 0.833 (0.782-0.884) | 1901 | 1821 | 80 | 21 (6-36) | 96 (95-97) | 6 (1-13) | 99 (99-99) | 0.035 | 0.013 |
| women | CoxPH | Black | 0.742 (0.638-0.847) | 1626 | 1537 | 89 | 34 (8-59) | 95 (94-96) | 5 (1-10) | 99 (99-100) | 0.035 | 0.007 |
| women | CoxPH | Other | 0.782 (0.738-0.827) | 3320 | 3147 | 173 | 32 (18-45) | 95 (95-96) | 10 (5-15) | 99 (99-99) | 0.035 | 0.016 |
| women | CoxPH | Lifelines | 0.781 (0.76-0.803) | 55697 | 51798 | 3899 | 23 (19-28) | 93 (93-93) | 5 (4-6) | 99 (99-99) | 0.035 | 0.014 |
| women | CoxGBT | White | 0.752 (0.746-0.758) | 214108 | 201213 | 12895 | 28 (26-29) | 94 (94-94) | 6 (6-7) | 99 (99-99) | 0.383 | 0.014 |
| women | CoxGBT | South Asian | 0.742 (0.697-0.787) | 2977 | 2789 | 188 | 28 (20-38) | 94 (93-95) | 9 (6-13) | 98 (98-99) | 0.383 | 0.02 |
| women | CoxGBT | Caribbean | 0.795 (0.751-0.84) | 2645 | 2459 | 186 | 30 (15-45) | 93 (92-94) | 6 (3-9) | 99 (99-99) | 0.383 | 0.013 |
| women | CoxGBT | East Asian | 0.825 (0.773-0.878) | 1901 | 1829 | 72 | 17 (4-31) | 96 (95-97) | 6 (1-13) | 99 (99-99) | 0.383 | 0.013 |
| women | CoxGBT | Black | 0.764 (0.668-0.86) | 1626 | 1545 | 81 | 34 (11-63) | 95 (94-96) | 5 (1-12) | 99 (99-100) | 0.383 | 0.007 |
| women | CoxGBT | Other | 0.77 (0.723-0.817) | 3320 | 3148 | 172 | 30 (18-43) | 95 (95-96) | 9 (5-14) | 99 (99-99) | 0.383 | 0.016 |
| women | CoxGBT | Lifelines | 0.785 (0.765-0.806) | 55697 | 52939 | 2758 | 18 (15-22) | 95 (95-95) | 5 (4-6) | 99 (99-99) | 0.383 | 0.014 |

Abbreviations: CI, confidence interval; PPV, positive predictive value; NPV, negative predictive value; CoxPH, Cox proportional hazards; CoxGBT, Cox gradient boosting.

# Questionnaire, measurement-based variables (or variables that require prior medical examination), and biomarkers

## Supplementary Figure S5A. Questionnaire, measurement-based variables (or variables that require prior medical examination), and biomarkers-based feature importance with coronary artery disease in women.

## Supplementary Figure S5B. Questionnaire, measurement-based variables (or variables that require prior medical examination), and biomarkers-based features’ hazard ratios for coronary artery disease prediction in women.

## Supplementary Figure S5C. Questionnaire, measurement-based variables (or variables that require prior medical examination), and biomarkers-based feature importance with coronary artery disease in men.

## Supplementary Figure S5D. Questionnaire, measurement-based variables (or variables that require prior medical examination), and biomarkers-based features’ hazard ratios for coronary artery disease prediction in men.

## Supplementary Table S11. Diagnostic metrics of the developed models containing questionnaire, measurement-based variables (or variables that require prior medical examination), and biomarkers.

| **Sex** | **Model** | **Ethnicity** | **C-index** | **N** | **Low risk** | **High risk** | **Sensitivity (95% CI)** | **Specificity (95% CI)** | **PPV (95% CI)** | **NPV (95% CI)** | **Threshold** | **10-year incidence** |
| --- | --- | --- | --- | --- | --- | --- | --- | --- | --- | --- | --- | --- |
| men | CoxPH | White | 0.711 (0.706-0.715) | 177301 | 130152 | 47149 | 57 (56-59) | 75 (74-75) | 8 (8-8) | 98 (98-98) | 0.044 | 0.037 |
| men | CoxPH | South Asian | 0.735 (0.708-0.762) | 3411 | 2544 | 867 | 55 (50-63) | 77 (75-78) | 14 (12-17) | 96 (95-97) | 0.044 | 0.066 |
| men | CoxPH | Caribbean | 0.723 (0.662-0.783) | 1538 | 1268 | 270 | 48 (31-61) | 83 (81-85) | 7 (4-10) | 98 (98-99) | 0.044 | 0.026 |
| men | CoxPH | East Asian | 0.662 (0.602-0.723) | 1572 | 1274 | 298 | 42 (25-57) | 82 (80-83) | 6 (3-8) | 98 (98-99) | 0.044 | 0.026 |
| men | CoxPH | Black | 0.746 (0.669-0.823) | 1577 | 1346 | 231 | 58 (43-73) | 86 (85-88) | 8 (5-10) | 99 (99-99) | 0.044 | 0.019 |
| men | CoxPH | Other | 0.731 (0.695-0.766) | 2779 | 2128 | 651 | 60 (50-69) | 78 (76-79) | 9 (7-10) | 98 (98-99) | 0.044 | 0.033 |
| men | CoxPH | Lifelines | 0.727 (0.71-0.744) | 39651 | 33167 | 6484 | 39 (36-42) | 85 (84-85) | 10 (9-10) | 97 (97-97) | 0.044 | 0.04 |
| men | CoxGBT | White | 0.703 (0.698-0.708) | 177301 | 130152 | 47149 | 56 (54-56) | 75 (74-75) | 8 (8-8) | 98 (98-98) | 0.268 | 0.037 |
| men | CoxGBT | South Asian | 0.717 (0.69-0.745) | 3411 | 2447 | 964 | 54 (48-60) | 74 (72-75) | 13 (11-15) | 96 (95-96) | 0.268 | 0.066 |
| men | CoxGBT | Caribbean | 0.721 (0.659-0.784) | 1538 | 1217 | 321 | 51 (36-69) | 80 (78-82) | 6 (4-9) | 98 (98-99) | 0.268 | 0.026 |
| men | CoxGBT | East Asian | 0.677 (0.615-0.739) | 1572 | 1235 | 337 | 54 (39-66) | 79 (77-82) | 7 (4-9) | 98 (98-99) | 0.268 | 0.026 |
| men | CoxGBT | Black | 0.746 (0.679-0.812) | 1577 | 1317 | 260 | 31 (14-45) | 84 (82-85) | 4 (2-6) | 98 (98-99) | 0.268 | 0.019 |
| men | CoxGBT | Other | 0.713 (0.677-0.749) | 2779 | 2106 | 673 | 57 (46-68) | 77 (76-78) | 8 (6-10) | 98 (98-99) | 0.268 | 0.033 |
| men | CoxGBT | Lifelines | 0.724 (0.706-0.741) | 39651 | 33286 | 6365 | 41 (37-45) | 85 (85-85) | 10 (9-11) | 97 (97-97) | 0.268 | 0.04 |
| women | CoxPH | White | 0.753 (0.747-0.76) | 214108 | 201214 | 12894 | 29 (27-30) | 94 (94-94) | 6 (6-7) | 99 (99-99) | 0.035 | 0.014 |
| women | CoxPH | South Asian | 0.757 (0.711-0.803) | 2977 | 2779 | 198 | 31 (19-39) | 94 (93-95) | 9 (5-13) | 99 (98-99) | 0.035 | 0.02 |
| women | CoxPH | Caribbean | 0.81 (0.764-0.855) | 2645 | 2493 | 152 | 30 (14-48) | 95 (94-95) | 7 (3-12) | 99 (99-99) | 0.035 | 0.013 |
| women | CoxPH | East Asian | 0.841 (0.792-0.89) | 1901 | 1828 | 73 | 17 (2-33) | 96 (95-97) | 6 (1-13) | 99 (99-99) | 0.035 | 0.013 |
| women | CoxPH | Black | 0.733 (0.622-0.843) | 1626 | 1536 | 90 | 34 (10-60) | 95 (94-96) | 5 (1-9) | 99 (99-100) | 0.035 | 0.007 |
| women | CoxPH | Other | 0.793 (0.748-0.838) | 3320 | 3138 | 182 | 39 (25-52) | 95 (94-96) | 11 (7-16) | 99 (99-99) | 0.035 | 0.016 |
| women | CoxPH | Lifelines | 0.777 (0.755-0.799) | 55697 | 52383 | 3314 | 20 (16-25) | 94 (94-94) | 5 (4-6) | 99 (99-99) | 0.035 | 0.014 |
| women | CoxGBT | White | 0.752 (0.746-0.758) | 214108 | 201214 | 12894 | 29 (27-30) | 94 (94-94) | 6 (6-7) | 99 (99-99) | 0.382 | 0.014 |
| women | CoxGBT | South Asian | 0.748 (0.704-0.793) | 2977 | 2808 | 169 | 27 (17-41) | 95 (94-95) | 10 (5-15) | 98 (98-99) | 0.382 | 0.02 |
| women | CoxGBT | Caribbean | 0.785 (0.737-0.834) | 2645 | 2503 | 142 | 24 (11-40) | 95 (94-96) | 6 (2-11) | 99 (99-99) | 0.382 | 0.013 |
| women | CoxGBT | East Asian | 0.838 (0.785-0.891) | 1901 | 1837 | 64 | 17 (4-30) | 97 (96-98) | 6 (1-14) | 99 (99-99) | 0.382 | 0.013 |
| women | CoxGBT | Black | 0.747 (0.64-0.853) | 1626 | 1551 | 75 | 34 (0-61) | 96 (95-97) | 5 (0-12) | 99 (99-100) | 0.382 | 0.007 |
| women | CoxGBT | Other | 0.776 (0.728-0.825) | 3320 | 3147 | 173 | 26 (15-36) | 95 (94-96) | 8 (4-12) | 99 (99-99) | 0.382 | 0.016 |
| women | CoxGBT | Lifelines | 0.782 (0.761-0.804) | 55697 | 53426 | 2271 | 15 (11-19) | 96 (96-96) | 5 (4-7) | 99 (99-99) | 0.382 | 0.014 |

Abbreviations: CI, confidence interval; PPV, positive predictive value; NPV, negative predictive value; CoxPH, Cox proportional hazards; CoxGBT, Cox gradient boosting.

# Risk stratification

## Supplementary Figure S6. Cumulative incidence of coronary artery disease by ethnicity and sex over time.

The x-axis represents years after baseline (initial assessment), while the y-axis indicates the cumulative CAD incidence. Data are stratified by sex (men and women) and population, including White, South Asian, Caribbean, East Asian, Black, Other, and Lifelines (external validation cohort). Cumulative incidence curves are plotted for low and high-risk groups according to QUES-CAD CoxPH and SCORE2 thresholds; for QUES-CAD we used the threshold that returned the same group size as SCORE2 in the White population. The lighter-colored lines represent the 95% CI. Abbreviations: CAD, coronary artery disease; QUES-CAD, Questionnaire-Based Evaluation for Estimating Coronary Artery Disease; CoxGBT, Cox gradient boosting; SCORE2, Systematic Coronary Risk Estimation 2; CI, confidence interval.
